# Multifunctional Film Assembled from N-Doped Carbon Nanofiber with Co–N_4_–O Single Atoms for Highly Efficient Electromagnetic Energy Attenuation

**DOI:** 10.1007/s40820-024-01440-2

**Published:** 2024-07-09

**Authors:** Jia Xu, Bei Li, Zheng Ma, Xiao Zhang, Chunling Zhu, Feng Yan, Piaoping Yang, Yujin Chen

**Affiliations:** 1 College of Materials Science and Chemical Engineering, Harbin Engineering University, Harbin, 150001 People’s Republic of China; 2 Key Laboratory of In-Fiber Integrated Optics, College of Physics and Optoelectronic Engineering, Harbin Engineering University, Harbin, 150001 People’s Republic of China

**Keywords:** Co single atoms, Asymmetric coordination structure, Axial oxygen coordination, Electromagnetic wave absorption, Multifunctional film

## Abstract

Single-atom materials have demonstrated attractive physicochemical characteristics. However, understanding the relationships between the coordination environment of single atoms and their properties at the atomic level remains a considerable challenge. Herein, a facile water-assisted carbonization approach is developed to fabricate well-defined asymmetrically coordinated Co–N_4_–O sites on biomass-derived carbon nanofiber (Co–N_4_–O/NCF) for electromagnetic wave (EMW) absorption. In such nanofiber, one atomically dispersed Co site is coordinated with four N atoms in the graphene basal plane and one oxygen atom in the axial direction. In-depth experimental and theoretical studies reveal that the axial Co–O coordination breaks the charge distribution symmetry in the planar porphyrin-like Co–N_4_ structure, leading to significantly enhanced dielectric polarization loss relevant to the planar Co–N_4_ sites. Importantly, the film based on Co–N_4_–O/NCF exhibits light weight, flexibility, excellent mechanical properties, great thermal insulating feature, and excellent EMW absorption with a reflection loss of − 45.82 dB along with an effective absorption bandwidth of 4.8 GHz. The findings of this work offer insight into the relationships between the single-atom coordination environment and the dielectric performance, and the proposed strategy can be extended toward the engineering of asymmetrically coordinated single atoms for various applications.

**Supplementary Information:**

The online version contains supplementary material available at 10.1007/s40820-024-01440-2.

## Highlights

- Asymmetrically coordinated Co–N_4_–O sites on N-doped carbon nanofiber were prepared.
- Co–O coordination along the axial direction led to enhanced dielectric polarization loss.
- Multifunctional films were developed for practical application in harsh environments.

**Supplementary Information:**

The online version contains supplementary material available at 10.1007/s40820-024-01440-2.

## Graphical abstract

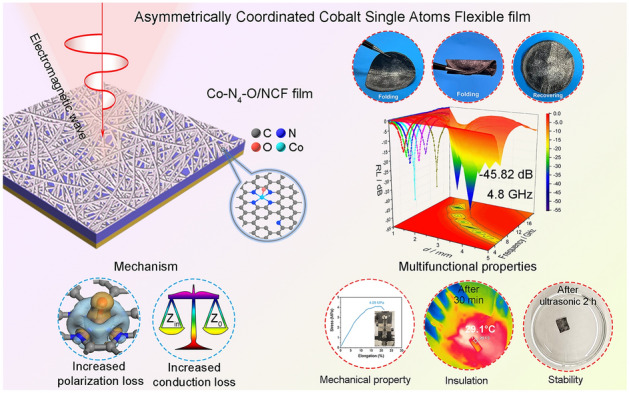

## Introduction

A widespread application of modern electronic devices based on gigahertz (GHz) electromagnetic signals has caused severe electromagnetic interference pollution, affecting human health and disturbing the regular operation of sophisticated electronic devices. In that regard, the design of high-performance electromagnetic wave (EMW) absorbing materials has been recognized as an efficient approach to diminish the hazards of electromagnetic radiation [1–4]. To date, various carbon nanomaterials, including graphene, carbon nanotubes and carbon nanofibers, have been developed to realize EMW absorption due to their low mass density, robust stability, and accessibility [5–8]. However, the lack of polarization sites and impedance mismatching caused by high electric conductivity endow the carbon nanomaterials with unsatisfactory EMW absorption properties, thereby greatly limiting their application range. Thus, developing high-performance EMW absorbers based on carbon nanomaterials remains a significant challenge.

In the last decade, single atoms anchored in a nitrogen-doped carbon matrix have attracted great attention from researchers because of nearly 100% atom utilization efficiency, extreme surface energy, and adjustable electronic structure [9–13]. In particular, unexpected EMW absorption properties have been discovered at atomically dispersed metal sites in the N-doped graphene (M–N_*x*_/G) [14–19]. Experimental results and theoretical calculations demonstrated that hybridization of the 3*d* orbitals of the transition metal atom and the 2*p* orbitals of the nitrogen atom allows for efficient charge transfer from the metal atom to the nitrogen atom, resulting in the formation of electrical dipoles. These electrical dipoles will induce polarization loss in the M–N_*x*_/G composite while being exposed to EMW irradiation, which will enhance the EMW absorption property of the material. In addition, the density of state (DOS) of the graphene is also increased upon the introduction of atomically dispersed transition metal atoms, which increases the electrical conductivity of the graphene matrix. Consequently, the conduction loss of the M–N_*x*_/G system is improved to a certain degree, contributing to the enhancement of EMW absorption property. Nevertheless, the transition-metal atom in the M–N_*x*_/G composite is frequently coordinated with four N atoms, so that the M–N_4_ moiety has a symmetrical planar structure. Such configuration significantly limits the further increase of the dielectric polarization loss of the M–N_*x*_/G system due to the symmetric distribution of the dipoles. Thus, there is significant room for improvement of the EMW absorption property of the M–N_*x*_/G.

Recent studies have demonstrated that the symmetric electronic structure of the M–N_4_ moiety can be broken by changing the kinds of coordinated nonmetallic atoms (O, S, P, B, etc.) at the first and/or second coordination spheres of the M–N_*x*_ structure [20–33]. Because of the difference between electronegativities of the N atom and other nonmetallic atoms, the symmetrically electronic structures of central metal single atoms can be adjusted to dramatically improve the activities of the carbon matrix containing the asymmetrically coordinated single atoms. For example, Hou et al. synthesized atomically dispersed nickel coordinated with nitrogen and sulfur species in porous carbon nanosheets for catalyzing the oxygen evolution reaction (OER) [34]. The density functional theory (DFT) calculations indicated that the sulfur atom tuned the hybridization states between Ni and the neighboring N atoms, which improved the local electronic structure of the Ni site and boosted the OER activity. Huang et al. fabricated asymmetric Ni–N_4_–O sites on porous graphitic carbons, using Mg(OH)_2_ as a sacrificed template for high-efficient CO_2_ electrocatalytic reduction reaction (CO_2_RR) [25]. Their in-depth experimental and theoretical results demonstrated that the axial Ni–O coordination broke the symmetric charge distribution, thereby reducing the Gibbs free energy for the rate-determining step of CO_2_RR relative to that of the planar Ni–N_4_ sites. Wan et al. constructed an atomic Co_1_–P_1_N_3_ interfacial structure in the ZIF-67-derived polyhedra, which exhibited much higher catalytic activity toward the hydrogen evolution reaction (HER) than the catalyst with a Co_1_–N_4_ interfacial structure [35]. According to theoretical calculations, charge depletion and charge accumulation on the P atoms and the surrounding carbon atoms provide more suitable hydrogen adsorption energy for the HER. Based on the above reports, the symmetric electronic structure of M–N_*x*_ can be broken by introducing other nonmetallic atoms, such as O, S, or P, which leads to the asymmetric charge distribution and formation of asymmetric electric dipoles. Compared to the symmetric electric dipoles, the asymmetric ones may produce more polarization loss upon EMW irradiation, which would be conducive to the electromagnetic wave absorption of the carbon matrix. However, the relationship between asymmetrically coordinated single atoms and the EMW absorbing performance of the carbon supports is in question. Moreover, regardless of various methods to construct asymmetrically coordinated single atoms, the precise control of their synthesis still faces a great challenge.

Herein, a facile water-assisted carbonization method was developed to synthesize well-defined asymmetrically coordinated Co–N_4_–O moieties with the axial O coordination using bacterial cellulose (BC) hydrogels as the precursor of the carbon nanofiber (Co–N_4_–O/NCF). The production of the Co–N_4_–O/NCF system consisted of the following steps. First, the Co ions were absorbed on the BC fiber (BC–Co^2+^), and the obtained BC–Co^2+^ structure was then carbonized in the presence of dicyandiamide (DCD) and subjected to acid etching. During the carbonization process, the BC fibers were transformed into carbon nanofibers for anchoring cobalt single atoms and nanoparticles, and DCD provided N coordination atoms for the single atoms to form a Co–N_4_ structure. Remarkably, the BC hydrogel contained abundant water, making it possible to produce water vapor to oxidize the Co atoms and achieve the axial Co–O coordination. Consequently, the precise synthesis of the well-defined asymmetrically coordinated Co–N_4_–O structure on NCF was successfully implemented through the current strategy. Moreover, in-depth experimental and theoretical studies revealed that doubly coordinated Co–N_4_–O moieties were favorable for increasing the dipole moment and polarization loss, which significantly boosted the EMW absorption property of carbon supports. As a result, the Co–N_4_–O/NCF film displayed an excellent EMW absorption property with a reflection loss of − 45.82 dB and an effective absorption bandwidth of 4.8 GHz. Meanwhile, the film possessed light weight, flexibility, outstanding mechanical properties, great thermal insulating features, and superior stability.

## Experimental Section

### Materials

Cobaltous chloride (CoCl_2_·6H_2_O, CAS No. 7791-13-1, ≥ 98.0% purity), acetic acid (CH_3_COOH, ≥ 99.0% purity) and sulfuric acid (H_2_SO_4_, ≥ 98.0% purity) were provided by Tianjin Guangfu Fine Chemical Research Institute, China. Dicyandiamide (DCD, C_2_H_4_N_4_, CAS No. 461-58-5, ≥ 99.0% purity) and sodium hydroxide (NaOH, CAS No. 1310-73-2, ≥ 99.0% purity) were provided by Sigma-Aldrich. Bacterial cellulose (BC) hydrogels were purchased from Hainan Yida Food Industry Co., Ltd., China. Paraffin wax was employed as the mixing substrate in the electromagnetic wave absorption experiments.

### Synthesis of NCF

To remove impurities, BC hydrogels were immersed in 0.02 M NaOH and 0.02 M CH_3_COOH solutions, respectively. After that, the purified BC hydrogels were washed with distilled water several times until a pH of 7.0 was achieved. The purified BC hydrogels were freeze-dried for over 48 h. After drying, BC and DCD taken in a weight ratio of 1: 20 were placed at the downstream and upstream sides of a tube furnace, respectively, and heated to 800 °C for 2 h at a heating rate of 5 °C min^−1^ under the Ar flow.

### Synthesis of Co–NPs/NCF

The purified BC hydrogels were mixed with 1.0 mM CoCl_2_ solution. Blends were then sonicated for 30 min and stirred for 6 h and finally cleaned in DI water three times. The as-obtained Co^2+^-BC hydrogels were freeze-dried for over 48 h. After drying, Co^2+^-BC and DCD with a weight ratio of 1: 20 were placed at the downstream and upstream sides of the tube furnace, respectively, and heated to 800 °C for 2 h at a heating rate of 5 °C min^−1^ under Ar flow. After that, the materials were cooled down naturally to room temperature and collected for use without any further treatment. The final product was referred to as Co–NPs/NCF.

### Synthesis of Co–N_4_–O/NCF

The Co–N_4_–O/NCF system was synthesized via the leaching of Co–NPs/NCF materials in 4.0 M H_2_SO_4_ solution at 80 °C for 5 h to remove Co NPs. The resulting product was collected via suction filtration, then washed with deionized water and ethanol several times, and finally dried at 60 °C.

### Synthesis of Co–N_4_/NCF

The NCF was mixed with 1.0 mM CoCl_2_ solution, and the mixture was sonicated for 30 min and stirred for 6 h. After that, the Co^2+^–NCF material was cleaned in DI water three times and freeze-dried for over 48 h. Subsequently, the sample was annealed in a tube furnace in an Ar atmosphere at 800 °C for 2 h at a heating rate of 5 °C min^−1^. At last, the Co–N_4_/NCF structure was synthesized via leaching at 80 °C for 5 h using 4.0 M H_2_SO_4_ solution. It was then washed with deionized water and ethanol several times via suction filtration and finally dried at 60 °C to remove Co NPs.

### Synthesis of Co–N_4_–O/NCF Film

First, 2 mL of KOH solution (75 mg mL^−1^) and 0.1 g of aramid nanofiber (ANF) were added to 50 mL of DMSO under continuous stirring at 50 °C for 3 h until the solution was dispersed homogeneously. Then, 20 mL of the above solution was dissolved in 20 mL of deionized water (hereinafter referred to as A solution), whereas 0.0115 g of Co–N_4_–O/NCF was dissolved in 10 mL of deionized water under continuous sonication (hereinafter referred to as B solution). Then, A and B solutions were mixed for 2 h until the final solution was dispersed homogeneously. This solution was filtrated and freeze-dried to remove residual moisture and to obtain the Co–N_4_–O/NCF film.

### Characterization Methods

The X-ray diffraction (XRD) experiments were carried out using an X'Pert Pro diffractometer equipped with a Cu-Kα radiation source (*λ* = 1.5418 Å) at a scan rate of 10° min^−1^. Scanning electron microscopy (SEM, Hitachi SU70), transmission electron microscopy (TEM, JEOL, JEM-2010) and aberration-corrected high-angle annular dark-field scanning transmission electron microscopy (AC-HAADF-STEM) analyses were performed to study the morphologies and microstructure of the samples. The X-ray photoelectron spectroscopy (XPS) measurements were taken using a PHI 5700 ESCA System. The Raman spectra were acquired by means of a Raman spectrometer (Xplora Plus, Horiba Jobin Yvon Lab) at the excitation wavelength of 532 nm. The X-ray absorption fine structure (XAFS) data was processed in Athena (version 0.9.26) software for background, pre-edge line, and post-edge line calibrations. The Fourier transform of the fitted XAFS spectra (FT-EXAFS) was afterward carried out in Artemis (version 0.9.26) software using the Co foil, Co_3_O_4_ and CoPc as references [29]. Four parameters, namely coordination number, bond length, Debye–Waller factor and *E*_0_ shift (CN, *R*, *σ*^2^, and Δ*E*_0_, respectively), were determined via fitting without being fixed, constrained, or correlated. The metal element contents in samples were assessed via inductively coupled plasma-optical emission spectrometry (ICP-OES, iCAP 7200 Series spectrometer). The mechanical tests of the Co–N_4_–O/NCF film were conducted using a Zwick Roell Z010 universal testing machine at a load speed of 20 mm min^−1^. The cylindrical samples with an inner diameter of 3.04 mm and an outer diameter of 7.00 mm were made by uniformly mixing 10 wt% absorber and 90 wt% paraffin wax. The electromagnetic parameters of the cylindrical samples were measured using a vector network analyzer (Anritsu MS4644A Vectorstar). After the Co–N_4_–O/NCF film was cut into cylindrical slices with the same dimensions, their electromagnetic parameters were examined using the same test procedure as before.

### Density Functional Theory Calculations

The DFT calculations in this study were carried out in Material Studio simulation environment where the electron exchange and the correlation energy were treated using generalized–gradient approximation (GGA) pseudopotentials and Perdew–Burke–Ernzerhof (PBE) exchange–correlation functional. The wave functions were expanded in a plane-wave basis set with a 450 eV energy cutoff. The convergences of energy and forces were 2 × 10^−5^ eV and 0.05 eV Å^−1^, respectively. A Monkhorst–Pack k-point grid with the dimensions of 3 × 3 × 1 was used for sampling of the first Brillouin zones of the surfaces for structural optimization. The C-N, Co–N_4_ and Co–N_4_–O models were constructed based on a graphene supercell containing a 4 × 4 optimized unit cell, as shown in Fig. S18. All atoms were fully relaxed during structural optimization. After structural relaxation, the differential charge density was calculated by means of the CASTEP module, and the dipole moment was executed using the Dmol3 module.

## Results and Discussion

### Composition and Structure

**Fig. 1:**
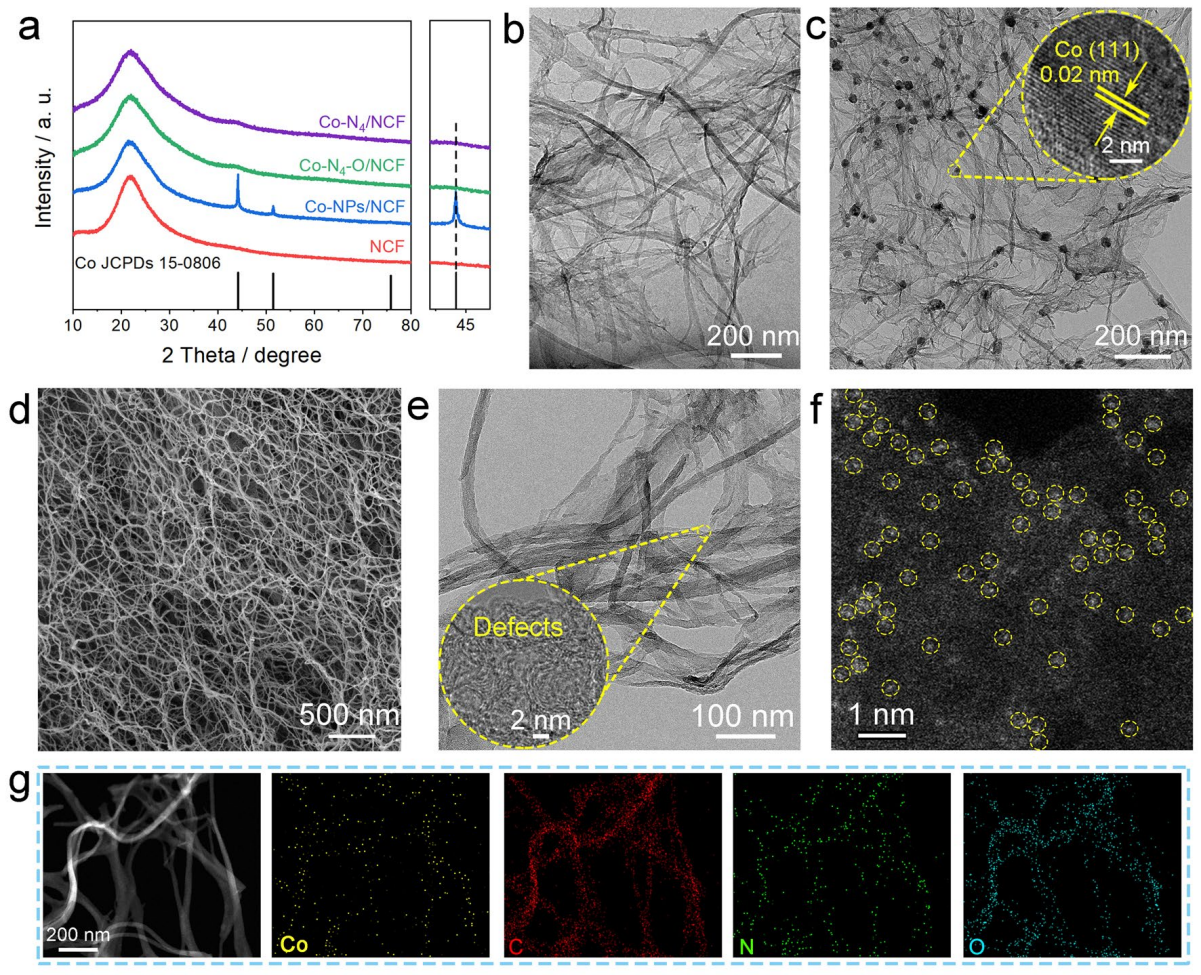
**a** X-ray diffraction patterns of NCF, Co–NPs/NCF, Co–N_4_–O/NCF, and Co–N_4_/NCF. TEM images of **b** NCF and **c** Co–NPs/NCF. **d** SEM, **e** TEM, and **f** AC–HAADF–STEM images of Co–N_4_–O/NCF. **g** HAADF–STEM image of Co–N_4_–O/NCF and corresponding EDS maps

The Co–N_4_–O/NCF material was synthesized through a water-assisted method. First, the purified BC hydrogel was immersed in a 1.0 mM CoCl_2_ solution for 6 h and then exposed to freeze-drying over 48 h. The dried Co ions-absorbed BC (BC–Co^2+^) and DCD with a weight ratio of 1: 20 were afterward placed at the downstream and upstream sides of the furnace, respectively, and annealed at 800 °C for 2 h under Ar flow. For convenience, the product obtained at this stage was labeled as Co–NPs/NCF. Subsequently, the Co–N_4_–O/NCF system was produced via acid etching of Co–NPs/NCF in 4.0 M H_2_SO_4_ solution at 80 °C for 5 h. For comparison, the NCF structure was synthesized through the same procedure as that of the Co–N_4_–O/NCF, except that there was without Co ion adsorption. Figure 1a depicts the XRD patterns of NCF, Co–NPs/NCF, and Co–N_4_–O/NCF. The XRD peaks at about 24° and 44° were assigned to the (002) and (101) planes of the carbon supports. Three additional peaks appeared at 44.2°, 51.5°, and 75.8° in the XRD patterns of Co–NPs/NCF, which were indexed to the (111), (200), and (220) planes of cubic Co (JCPDS#15–0806, Fmm(225) space group), respectively. However, these peaks were absent in the XRD pattern of the Co–N_4_–O/NCF specimen (see Fig. 1a and magnified XRD spectrum at 45°), suggesting that the Co NPs were removed by acid etching.

According to the SEM analysis (Fig. S1), the carbon nanofibers in the NCF sample crossed each other and their length was about several tens of microns. The TEM image (Fig. 1b) revealed that the carbon nanofibers had smooth surfaces and their diameters were 20–50 nm. After the loading of Co NPs, there was a slight change in the size and cross-linked structure of carbon nanofibers (see the SEM images in Fig. S2). The inset in Fig. 1c shows an interplanar spacing of 0.20 nm, consistent with that of a cubic Co(111) plane. The diameter of Co NPs anchored to the carbon nanofibers was approximately 24 nm (Fig. S3). The HAADF-STEM image and the corresponding energy-dispersive X-ray spectroscopy (EDS) map further confirmed that Co nanoparticles were anchored on the carbon nanofiber surface (Fig. S4). After acid leaching, the obtained Co–N_4_–O/NCF specimen kept the interconnected network structure. Meanwhile, the surface of carbon nanofiber became smooth again, exhibiting neither nanoparticles nor clusters atop (Fig. 1d). This revealed that the Co NPs were removed entirely from the carbon nanofibers. The inset in Fig. 1e displays the curved interplanar fringes of the graphitic carbon, indicating the existence of abundant defects in the Co–N_4_–O/NCF sample. The formation of abundant defects was favorable to the high dielectric loss upon electromagnetic wave radiation, which improved the EMW absorption property of the Co–N_4_–O/NCF sample. To further gather detailed information on the Co distribution in the Co–N_4_–O/NCF sample, the AC-HAADF-STEM was applied. Figure 1f depicts numerous bright dots marked with yellow circles in the AC-HAADF-STEM image, which corresponded to heavy Co atoms, implying that Co sites were atomically dispersed in the carbon nanofibers. As seen from the HAADF-STEM and corresponding EDS maps, Co, C, N, and O elements were uniformly distributed in the carbon nanofibers. Nevertheless, the Co-, C-, N-, and O-related signals were mostly overlapped, suggesting that the Co atoms might be coordinated with N or O (Fig. 1g). The ICP-OES data indicated that the Co contents in the Co-NPs/NCF and Co–N_4_–O/NCF specimens were 9.54 and 1.34 wt%, respectively.

The XPS experiments were conducted to identify the chemical components in the NCF, Co–NPs/NCF, and Co–N_4_–O/NCF samples. In particular, the survey XPS spectra revealed the presence of Co, C, N, and O species in the Co–NPs/NCF and Co–N_4_–O/NCF specimens (Fig. S5). In the Co 2*p* XPS spectrum of the Co–NPs/NCF system, the peak at 778.3 eV was assigned to metallic Co, while those at 780.5 and 796.1 eV were attributed to Co 2*p*_3/2_ and Co 2*p*_1/2_ oxidation states of Co as a result of the contact of the samples with air (Fig. S6) [36]. In contrast, no peak corresponding to Co^0^ species was observed in the Co 2*p* XPS spectrum of the Co–N_4_–O/NCF sample. In the meanwhile, the Co-related peaks in the Co–N_4_–O/NCF materials were ascribed to the Co^δ+^ (2 < δ < 3) states (Fig. S6). In the C 1*s* XPS spectra of the three samples, the peaks resolved at 284.6, 285.4, 286.5, and 288.7 eV were ascribed to C–C, C–N, C–O, and C–O–O–R species, respectively (Fig. S7) [37]. The N 1*s* XPS spectra of the Co–N_4_–O/NCF and Co–NPs/NCF specimens were deconvolved into four peaks with the binding energies of 401.2, 400.1, 399.2, and 398.2 eV, which were associated with graphitic–N, pyrrolic–N, Co–N_*x*_, and pyridinic–N moieties, respectively (Fig. S8b, c) [38]. The peaks between 403.0 and 406.0 eV can be attributed to N–O bonds [39]. In contrast, only graphitic–N, pyridinic–N and pyrrolic–N were detected in the N 1*s* XPS spectrum of the NCF sample (Fig. S8a). According to the XPS analysis, the N contents in the NCF, Co–NPs/NCF, and Co–N_4_–O/NCF samples were approximately 2.61, 6.42, and 4.95 at%, respectively. Among these N species, the Co–N_*x*_ concentrations in the Co–NPs/NCF and Co–N_4_–O/NCF were about 5.83 and 15.52 at%, respectively (Fig. S9). The presence of the Co–N_*x*_ species in the Co–NPs/NCF and Co–N_4_–O/NCF materials implied the atomically dispersed Co sites in two samples. In the O 1*s* XPS spectra (Fig. S10), the Co–O bonds at 529.8 eV were identified in the Co–N_4_–O/NCF samples in addition to the O–C–O and C–OH species, implying that the Co species in the Co–N_4_–O/NCF structure might be coordinated with the O atoms. The Raman spectra of the NCF, Co–NPs/NCF, and Co–N_4_–O/NCF materials exhibited the characteristic peaks of carbonaceous materials, namely D band at 1350 cm^–1^ (a disorder-induced phonon mode) and G band at 1590 cm^–1^ (an intrinsic in-plane vibration of *sp*^2^ carbon atoms in graphite) (Fig. S11) [40]. The D-to-G intensity ratios (*I*_D_/*I*_G_) of the NCF, Co–NPs/NCF, and Co–N_4_–O/NCF were 0.84, 0.87, and 1.07, respectively. The higher ratio value of the Co–N_4_–O/NCF specimen indicated that it had more carbon defects than other samples.

**Fig. 2:**
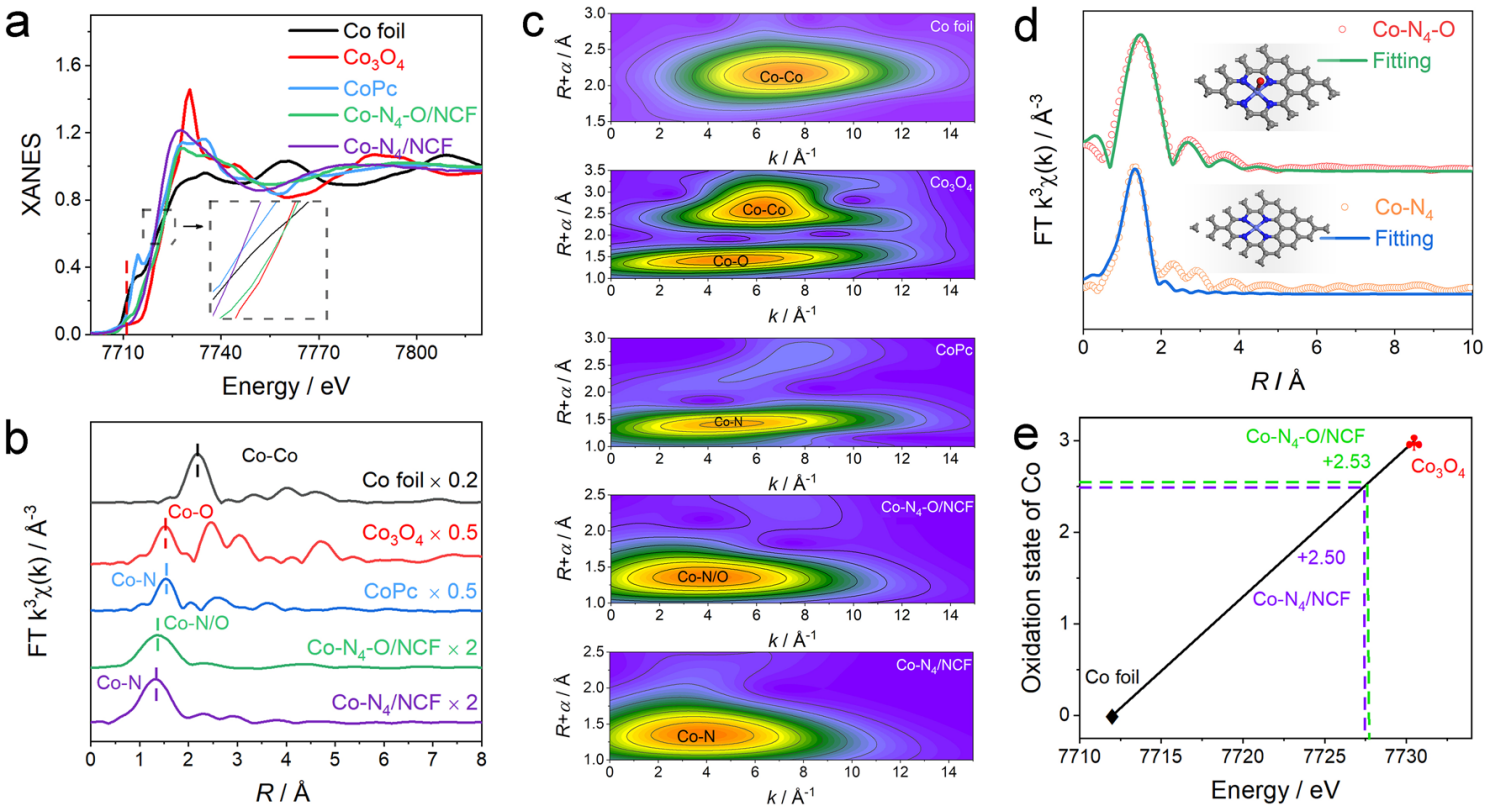
**a** XANES spectra and **b** FT–EXAFS curves of Co foil, Co_3_O_4_, CoPc, Co–N_4_–O/NCF, and Co–N_4_/NCF at Co K–edge. **c** WT–EXAFS plots of Co foil, Co_3_O_4_, CoPc, Co–N_4_–O/NCF, and Co–N_4_/NCF. **d** The corresponding EXAFS fitting curves of Co–N_4_–O/NCF and Co–N_4_/NCF with configurations in *k* space. The insets are the schematic models of Co–N_4_–O and Co–N_4_ configurations. **e** State oxidation of Co determined from the Co K-edge XANES data

To further analyze the electronic state and coordination environment of Co single atoms in the Co–N_4_–O/NCF sample, X-ray absorption near-edge structure (XANES) and extended X-ray absorption fine structure (EXAFS) measurements were taken. Figure 2a depicts the Co near-edge absorption energy spectra of Co–N_4_–O/NCF, and the reference samples. The Co K-edge XANES spectra of Co–N_4_–O/NCF were located between those of the Co foil and Co_3_O_4_, implying that Co single atoms in both specimens were positively charged. Furthermore, the Co–N_4_–O/NCF had a similar near-edge structure to that of CoPc with some differences in various energy regions, suggesting that the Co single atoms therein were coordinated with N atoms by analogy with CoPc. It is noteworthy that the XANES data of CoPc (the black line) revealed the presence of a pre-edge peak at ~ 7710 eV, which was the fingerprint of a Co–N_4_ square-planar structure [41, 42]. However, the magnified pre-edge peak in the spectrum of Co–N_4_–O/NCF implied that the centrosymmetry of Co sites was distorted [43]. In the inset of Fig. 2a, a drastic decrease in the peak of the Co–N_4_–O/NCF structure was ascribed to a defective graphene architecture around the Co atoms [44]. The structures of Co single atoms in the Co–N_4_–O/NCF specimens were further corroborated by the Fourier-transformed (FT) *k*^3^-weighted EXAFS analysis (Fig. 2b). The dominant peak at ~ 1.36 Å could be assigned to the Co–N/O structure, while the absence of the feature corresponding to the Co–Co scattering (~ 2.18 Å) further confirmed the atomic dispersion of Co species in the Co–N_4_–O/NCF sample. The wavelet transform (WT) of the Co K-edge EXAFS data was carried out to elucidate the structure of single Co atoms in the Co–N_4_–O/NCF system (Fig. 2c) [30, 45]. The WT contour plots of both specimens exhibited one intensity maximum at 3.9 Å^−1^, which was close to that of CoPc. Unlike the WT plots of Co foil, Co_3_O_4_ and CoPc, the lack of the intensity maximum from the Co–Co contribution further indicated that Co species were atomically dispersed in the Co–N_4_–O/NCF sample. In addition, the quantitative structural parameters of Co atoms in Co–N_4_–O/NCF were afterward extracted via the EXAFS fitting in both *R* and *k* spaces. The fitting parameters (Table S1) and the fitting curves (Fig. S12) suggested the Co–N shell with a coordination number (CN) of 4 at a distance of 1.97 Å and the Co–O shell with a CN of 1 at a distance of 1.71 Å. The geometrical configuration of the Co atom based on the DFT calculations is depicted in the inset of Fig. 2d, in which one isolated cobalt atom in the NCF was coordinated with four N atoms to form a planar Co–N_4_ structure, whereas another one was coordinated with one O atom along the axial direction of the plane. Unlike other synthetic methods, the BC hydrogel precursor in this work contained a large amount of water, which generated water vapor during the carbonization process and could in situ oxidize the Co atoms to form Co–N_4_–O moieties. Thus, the proposed water-assistant carbonization strategy can be extended to produce other asymmetric M–N_4_–O sites on the carbon nanofibers.

**Fig. 3:**
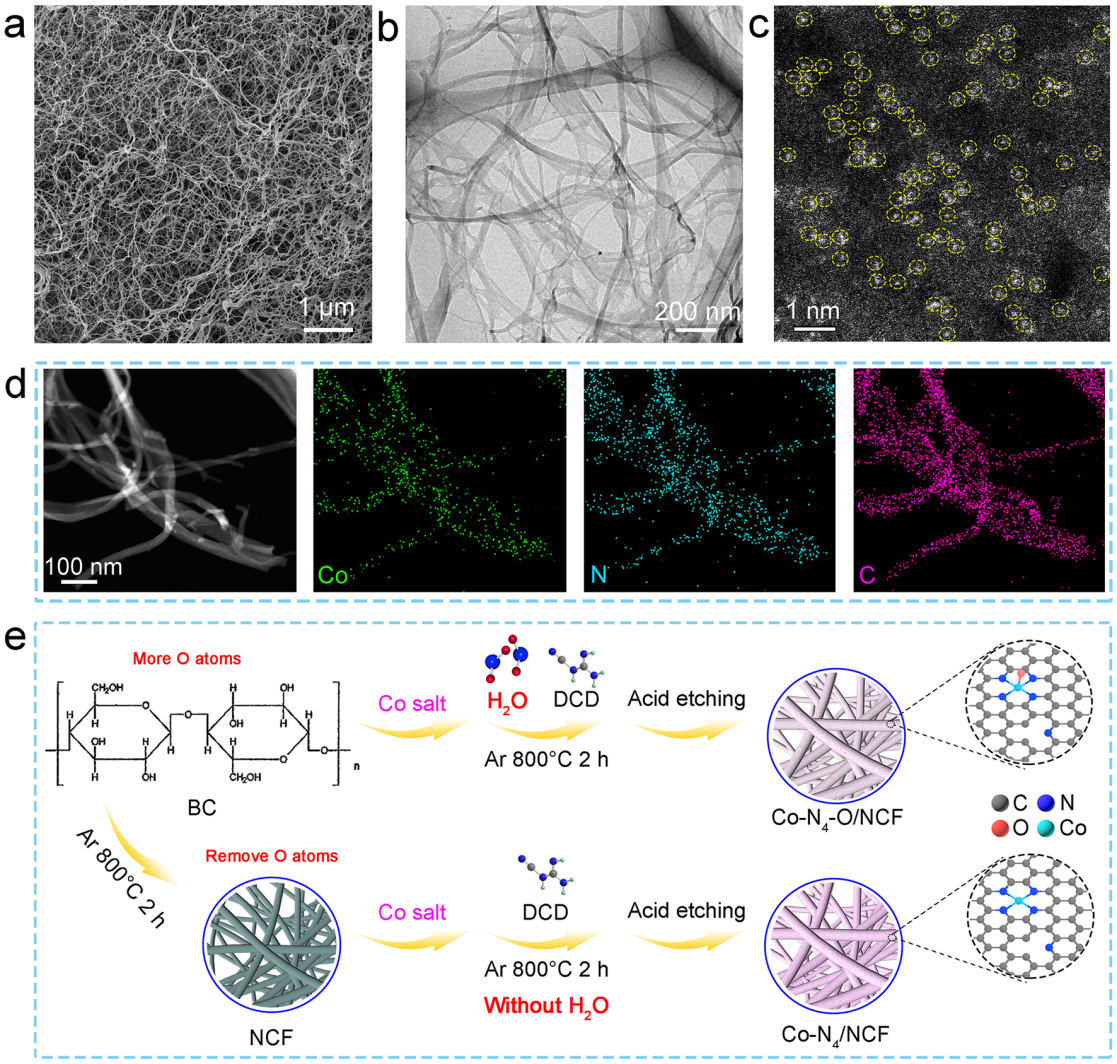
**a** SEM, **b** TEM, and **c** AC–HAADF–STEM images of Co–N_4_/NCF. **d** HAADF–STEM image of Co–N_4_/NCF specimen and corresponding EDS maps. **e** Schematic of synthesis mechanisms for Co–N_4_–O/NCF and Co–N_4_/NCF

To provide an insight into the synthesis mechanism of the water-assistant carbonization strategy, the Co–N_4_/NCF sample was synthesized through the pre-carbonization route to remove the O atoms (see Sect. 2). The diffraction peaks of the Co–N_4_/NCF sample in the XRD pattern were similar to those of Co–N_4_–O/NCF (Fig. 1a). Besides, the SEM, TEM, HAADF-STEM, and EDS maps revealed that the Co–N_4_/NCF composite consisted of carbon nanofibers with uniform diameters and homogeneously distributed Co, C and N elements therein (Figs. 3a–d and S13). In the AC-HAADF-STEM image of Co–N_4_/NCF (Fig. 3c), numerous bright dots (marked with yellow circles) corresponded to heavy Co atoms, implying that Co sites were atomically dispersed in Co–N_4_/NCF. The ICP-OES data indicated that the Co content in the Co–N_4_/NCF sample was 1.39 wt%. Meanwhile, according to the XPS analysis, the Co 2*p* peaks of the Co–N_4_/NCF specimen exhibited a slight shift toward the lower binding energies relative to those of the Co–N_4_–O/NCF, which indicated the lower oxidation state of Co in the former specimen (Fig. S6). The N contents and the Co–N_*x*_ concentrations in the Co–N_4_/NCF sample were approximately 13.74 at% (Fig. S9), being close to those in the Co–N_4_–O/NCF. Besides, the O 1*s* XPS spectra of the Co–N_4_/NCF sample exhibited the absence of Co–O bonds (Fig. S10), suggesting that Co species might have not been coordinated with the O atoms in the Co–N_4_/NCF. The Raman spectra of the Co–N_4_/NCF specimen included the same characteristic peaks of carbonaceous materials, and the corresponding *I*_D_/*I*_G_ ratio was 1.07 (Fig. S11).

To determine the electronic state and coordination environment of Co single atoms in the Co–N_4_/NCF sample, XANES and EXAFS spectra from Co–N_4_–O/NCF and Co–N_4_/NCF specimens were afterward compared. As seen from the Co K-edge positions at the half of the maximum edge intensity, it was found that the valence state of Co in Co–N_4_/NCF was lower than that in Co–N_4_–O/NCF, which was consistent with the Co 2*p* XPS results. The FT *k*^3^-weighted EXAFS analysis (Fig. 2b) and the WT of the Co K-edge EXAFS (Fig. 2c) data revealed that the electronic state and coordination environment of the Co–N_4_/NCF configuration were close to those of CoPc, indicating that the Co species were atomically dispersed in the Co–N_4_/NCF. In addition, the quantitative structural parameters of Co atoms in Co–N_4_/NCF were afterward extracted via the EXAFS fitting in both *R* and *k* spaces. The fitting parameters (Table S1) and the fitting curves (Fig. S14) suggested the fitting parameters and the fitting curves of Co–N_4_/NCF revealed that one Co atom was coordinated with four N atoms without the formation of a Co–O shell. According to Fig. 2e, the oxidation states of Co were + 2.53 and + 2.50 in Co–N_4_–O/NCF and Co–N_4_/NCF, respectively, meaning that the valence state of Co in the Co–N_4_–O/NCF was slightly higher than that in the Co–N_4_/NCF, which was consistent with the XPS results. Therefore, the experimental results demonstrated that the method under consideration enabled one to synthesize the asymmetric Co–N_4_–O moiety rather than the symmetric planar Co–N_4_ configuration on the carbon nanofibers. The synthesis mechanism of the Co–N_4_–O/NCF through the water-assistant carbonization route is shown in Fig. 3e. Unlike other synthetic methods, the BC hydrogel precursor in this work contained a large amount of water, which generated water vapor during the carbonization process and could in situ oxidize the Co atoms to form Co–N_4_–O moieties. Thus, the proposed water-assistant carbonization strategy can be extended to the synthesis of other asymmetric M–N_4_–O sites on the carbon nanofibers.

### Electromagnetic Energy Attenuation Performance and Mechanism

**Fig. 4:**
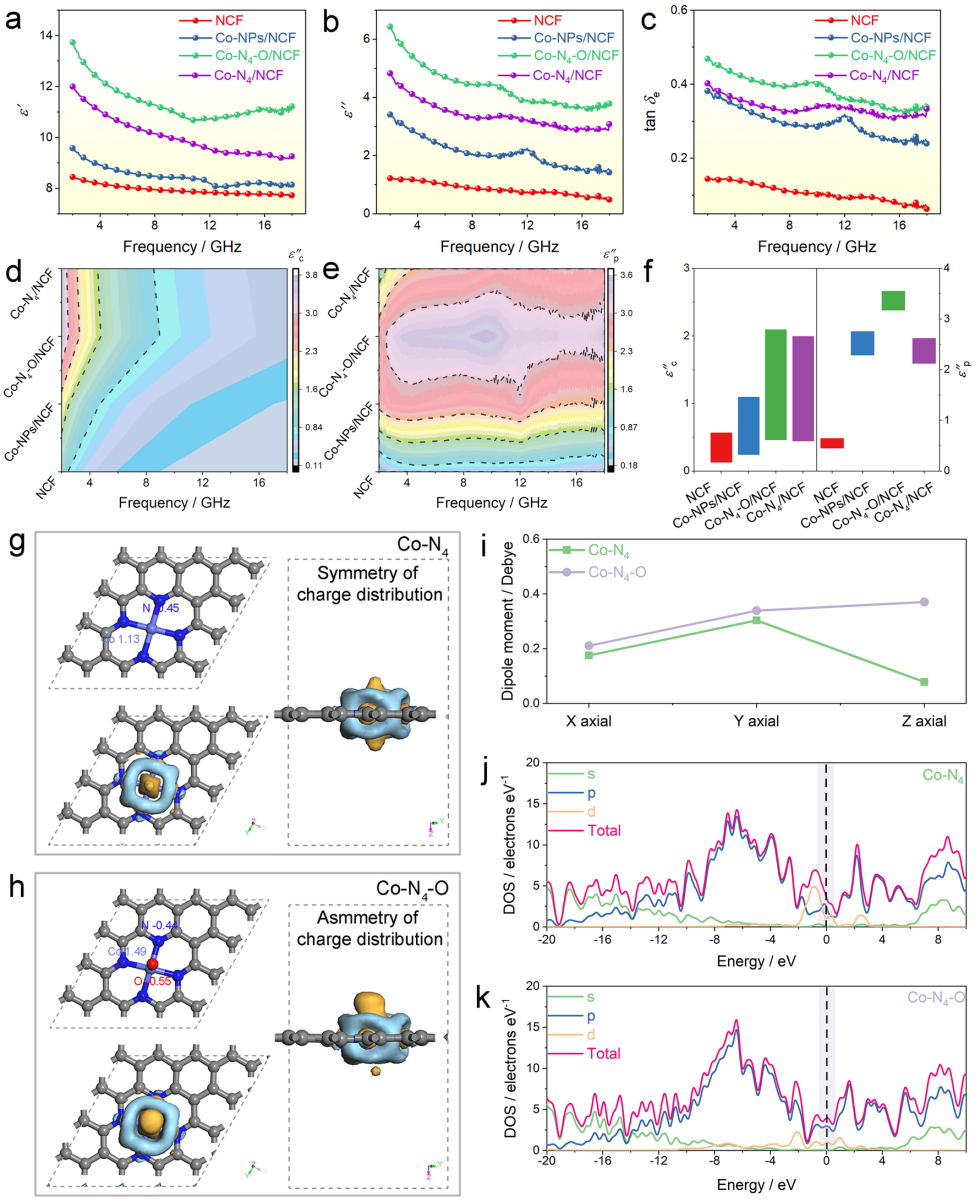
**a** *ε′*, **b** *ε*″, **c** tan *δ*_e_, **d** *ε*_c_″, and **e** *ε*_p_″ of NCF, Co–NPs/NCF, Co–N_4_–O/NCF, and Co–N_4_/NCF. **f** *ε*_c_″ and *ε*_p_″ of NCF, Co–NPs/NCF, Co–N_4_–O/NCF, and Co–N_4_/NCF. The Mulliken population distributions and the charge density difference of **g** Co–N_4_ and **h** Co–N_4_–O. Here, the blue and yellow colors represent charge depletion and charge accumulation, respectively. The isosurfaces refer to the isovalue of 0.015 e Å^−3^. The C, N, O and Co atoms are represented by the gray, blue, red and aquamarine colors, respectively. **i** Dipole moment values of Co–N_4_ and Co–N_4_–O. The calculated projected density of states (DOSs) of **j** Co–N_4_ and **k** Co–N_4_–O. (Color figure online)

Because of asymmetric cobalt single atoms and the high aspect ratio of carbon nanofibers, the Co–N_4_–O/NCF sample might have good EMW absorption properties. To verify this, the electromagnetic parameters of the NCF, Co–NPs/NCF, Co–N_4_–O/NCF, and Co–N_4_/NCF with 10 wt% mass loadings in the paraffin matrix were measured using a vector network analyzer (Anritsu MS4644A Vectorstar). The parameters included the relative complex permittivity (*ε*_r_ = *ε*′* − j ɛ*″) and permeability (*μ*_r_ = *μ*′ − *j μ*″). The real part (*ε*′) and the imaginary part (*ɛ*″) of the permittivity reflected the storage and attenuation capability of electromagnetic energy, respectively. The four samples showed very low real part (*μ*′) and imaginary part (*μ*″) values of the permeability (Fig. S15). As shown in Fig. 4a, b, the *ε*′ and *ɛ*″ values of the three samples decreased with the increase in the frequency from 2 to 18 GHz. Among the four samples, the NCF exhibited the lowest *ε*′ (8.44–7.73) and *ɛ*″ (1.21–0.48) levels (Fig. 4a, b), indicating the inferior electromagnetic energy storage and attenuation capabilities. After the introduction of Co NPs, the *ε*′ and *ɛ*″ values of the Co–NPs/NCF were found to increase significantly, meaning that the Co NPs could have enhanced the dielectric loss of the NCF (Fig. 4a, b). Both the *ε*′ and *ɛ*″ parameters of the Co–N_4_/NCF were larger than those of the other two samples (Fig. 4a, b), implying that the presence of a sole Co–N_4_ structure was conducive to the improvement of the dielectric performance of the NCF support. It is noteworthy that Co–N_4_–O/NCF had a better dielectric performance than Co–N_4_/NCF. Meanwhile, the dielectric loss (tan *δ*_ε_) of the Co–N_4_–O/NCF ranged from 0.34 to 0.47, which was also more prominent than those of the NCF, Co–NPs/NCF, and Co–N_4_/NCF (Fig. 4c). In a word, the Co–N_4_–O/NCF system possessed a significantly enhanced dielectric loss property. In the gigahertz frequency range, the dielectric loss (*ε*″) mainly included the conduction loss (*ε*_c_″) and the polarization loss (*ε*_p_″). The semicircles and the long tails in the Cole–Cole plots of the NCF, Co–NPs/NCF, Co–N_4_–O/NCF and Co–N_4_/NCF exhibited the contribution from both kinds of losses (Fig. S16). As for the *ε*_c_″ component, it was calculated according to formulae (1–3) in the Supporting Information [46]. As shown in Fig. S17, the electrical conductivities (*σ*) of the NCF, Co–NPs/NCF, and Co–N_4_–O/NCF cylindrical samples were measured to be 0.13, 0.22, 0.42, and 0.40 S m^−1^, respectively. In that regard, the *ε*_c_″ values varied according to a sequence of Co–N_4_–O/NCF > Co–N_4_/NCF > Co–NPs/NCF > NCF (Fig. 4d). Based on the *ε*_c_″ values, the *ε*_p_″ ones of the four samples could be estimated (Fig. 4e). It was found that the *ε*_p_″ parameters of the four specimens followed the same order as that of the *ε*_c_″ one. Thus, the increase in dielectric loss of the Co–N_4_–O/NCF sample was attributed to the enhancement of both conduction and polarization losses (Fig. 4f).

Based on the FT-EXAFS and WT-EXAFS data, there were the asymmetrical well-defined Co–N_4_–O sites in the Co–N_4_–O/NCF sample, whose electronic structure differed from that of the symmetric Co–N_4_. To analyze the effect of the coordination environment of the Co single atoms on the dielectric loss, DFT calculations were implemented. The models of the C–N, Co–N_4_, and Co–N_4_–O configurations were established conforming to the structural characterization results (Fig. S18). Figures 4g, h S19a and Table S2 display the Mulliken population distributions of the optimized C–N, Co–N_4_, and Co–N_4_–O configurations. In the C–N configuration, the charge of N species was − 0.34 (Fig. S19a). The charges of Co species in Co–N_4_ and Co–N_4_–O configurations were found to be 1.13 and 1.49, respectively. The higher positive charge density around the Co atoms in the Co–N_4_–O moieties indicated the faster electron transfer from Co toward N and O relative to that in the Co–N_4_. The difference between the charge transfer rates has broken the planar configuration symmetry, which is evidenced by the differential charge density diagram for the Co–N_4_ and Co–N_4_–O configurations (Fig. 4g, h). The more positive charges of Co sites and asymmetric charge distributions in the Co–N_4_–O moiety resulted in a stronger electric dipole polarization, leading to the enhanced dielectric loss of the Co–N_4_–O/NCF sample. The dipole moments of the Co–N_4_ and Co–N_4_–O configurations were calculated to further understand the mechanism of dielectric loss performance. As shown in Figs. 4i, S19b and Table S3, the dipole moments of the Co–N_4_–O moiety along the *x*-,* y*- and *z*-axis were remarkably larger than those of the planar Co–N_4_ and C–N, indicating the enhanced polarizability of Co–N_4_–O moieties. In particular, the dipole moment of the Co–N_4_–O moiety along the *z*-axis, i.e., the same direction of the axial Co–O band, was ~ 5 times that of the planar Co–N_4_ structure (Fig. 4i and Table S3). Thus, the introduction of the axial O coordination could noticeably improve the dielectric loss of the Co–N_4_ structure. The projected density of states was further computed to elucidate the effect of asymmetric Co–N_4_–O moieties on the conduction loss of the Co–N_4_–O/NCF sample. According to Figs. 4j, k and S19c, the DOS of the Co–N_4_–O configuration was higher than that of the Co–N_4_ and C–N near the Fermi level, suggesting the increase in electrical conductivity of the Co–N_4_–O configuration. Consequently, the Co–N_4_–O structure exhibited a larger conduction loss than Co–N_4_. The above DFT calculations revealed that the incorporation of the axial O coordination could have broken the symmetry of the electronic structure of the planar Co–N_4_ structure, thereby improving its polarizability and conductivity. This explained the enhanced dielectric loss of the Co–N_4_–O/NCF sample compared to the counterparts with the planar Co–N_4_ sites. As previously reported, incorporating the Co–N_4_ moieties into the N-doped carbon matrix could improve both the conduction and polarization losses [14, 16]. Thus, the dielectric loss of the Co–N_4_–O/NCF system exceeded that of the NCF support. Notably, the Co–NPs/NCF material also contained the Co–N_4_–O sites; however, it exhibited a lower dielectric loss than the Co–N_4_–O/NCF one. According to the TEM and ICP analyses, the Co–NPs/NCF had additional Co NPs concerning the Co–N_4_–O/NCF sample. As a result, the number of carbon nanofibers in the Co–N_4_–O/NCF/paraffin composite was approximately 8.20 wt% greater than that in the Co–NPs/NCF/paraffin system at the same mass loading. Meanwhile, there were also abundant Co single atoms in the Co–N_4_–O/NCF/paraffin composite. Besides that, excessive carbon nanofibers were favorable to the formation of a conductive network, leading to the increase in conduction loss of the Co–N_4_–O/NCF sample. On the other hand, more Co–N_4_–O sites could induce stronger polarization, increasing polarization loss of the Co–N_4_–O/NCF sample. Thus, it was reasonable that the Co–N_4_–O/NCF had a higher dielectric loss than the Co–NPs/NCF. Based on the experimental data and theoretical calculations, the enhanced dielectric loss of the Co–NPs/NCF specimen was attributed to the increase in the conduction and polarizations owing to the asymmetric coordination environment of the Co–N_4_–O moieties as well as the sufficient amount of the conductive NCF support and the Co single atoms in the paraffin matrix.

**Fig. 5:**
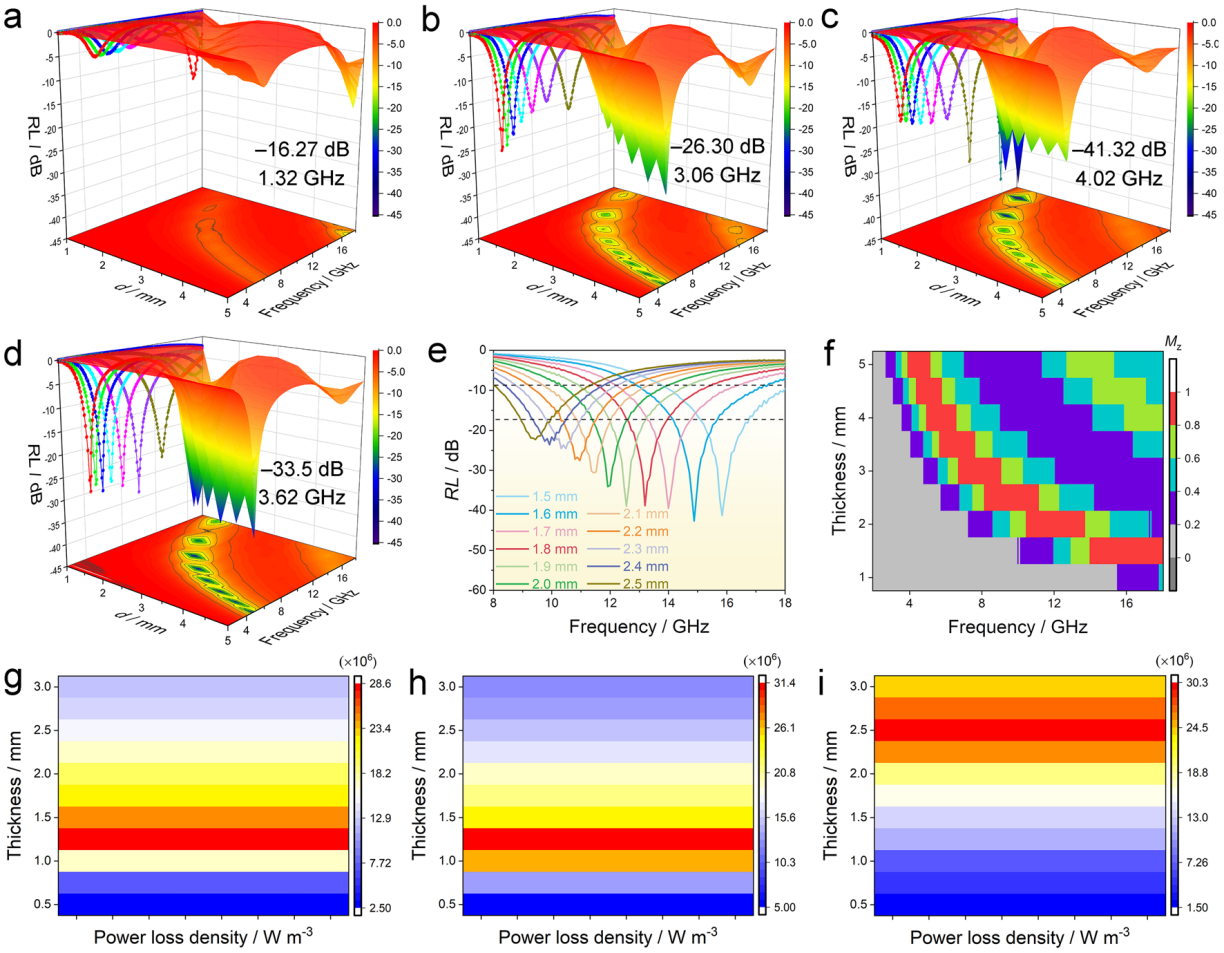
3D representation of *RL* values of **a** NCF, **b** Co–NPs/NCF, **c** Co–N_4_–O/NCF, and **d** Co–N_4_/NCF. **e** *RL*–*f* dependences of Co–N_4_–O/NCF at the matching thickness of 1.5–2.5 mm. The power loss densities of **g** Co–NPs/NCF, **h** Co–N_4_–O/NCF, and **i** Co–N_4_/NCF

The EMW absorption properties of the NCF, Co–NPs/NCF, Co–N_4_–O/NCF, and Co–N_4_/NCF samples were assessed from the reflection loss (*RL*) values according to the transmission line theory [46]. Among the four samples, the NCF one showed the worst EMW absorption property with a minimal *RL* (*RL*_min_) value of − 16.27 dB and an effective absorption bandwidth (EAB, *RL* ≤  − 10 dB) of only 1.32 GHz at the matching thickness (*d*) of 5.0 mm (Fig. 5a). After introducing the Co NPs, the EMW absorption property of the NCF matrix was improved significantly. For example, the *RL*_min_ and EAB values of the Co–NPs/NCF reached − 26.30 dB and 3.06 GHz at *d* of 5.0 mm, respectively (Fig. 5b). In Fig. 5d, the *RL*_min_ and EAB of the Co–N_4_/NCF at the *d* of merely 1.5 mm were − 33.5 dB and 3.62 GHz, respectively. Furthermore, the *RL*_min_ and EAB parameters of the Co–N_4_–O/NCF composite sharply increased to − 41.32 dB and 4.02 GHz at *d* of 1.5 mm, respectively (Fig. 5c). As for the Co–N_4_–O/NCF system, its *RL*_min_ values almost exceeded − 20 *d*B at the *d* values in the range of 1.5–5.0 mm, meaning that the sample could attenuate 99% of the EMW energy. Moreover, the *RL*_min_ values of the specimen exceeded − 30 dB even at the matching thickness between 1.5 and 1.8 mm (Fig. 5e). Therefore, in view of the *RL* and EAB, the Co–N_4_–O/NCF structure exhibited a remarkable enhancement of the EWM absorption properties, especially at a low matching thickness. In addition, the composite possessed the relatively large attenuation constant (*α*) values (54.73–310.81) calculated according to Eq. (S6) in the Supporting Information, which were superior to those of NCF (43.32–191.07), Co–NPs/NCF (14.59–76.43), and Co–N_4_/NCF (38.68–267.81), further confirming the best attenuation ability (Fig. S20). Since the EMW absorption properties among the four samples followed the same order of the relevant dielectric losses, the enhanced EMW absorption of the Co–N_4_–O/NCF system could be explained by the higher dielectric loss. Besides that, the specimen had better impedance matching characteristics in terms of the impedance matching degree (*M*_z_), as shown in Figs. 5f and S21. The improvement in impedance matching could ensure more incident EMW into the absorbing samples, endowing the Co–N_4_–O/NCF system with outstanding EMW absorption performance. Moreover, in view of *RL*, EAB, *d,* and filler mass, the Co–N_4_–O/NCF material exhibited the better performance compared to other recently reported absorbers, indicating that it could be an ideal absorber for the actual applications in the EMW absorption (Table S4) [16–18, 47–60].

**Fig. 6:**
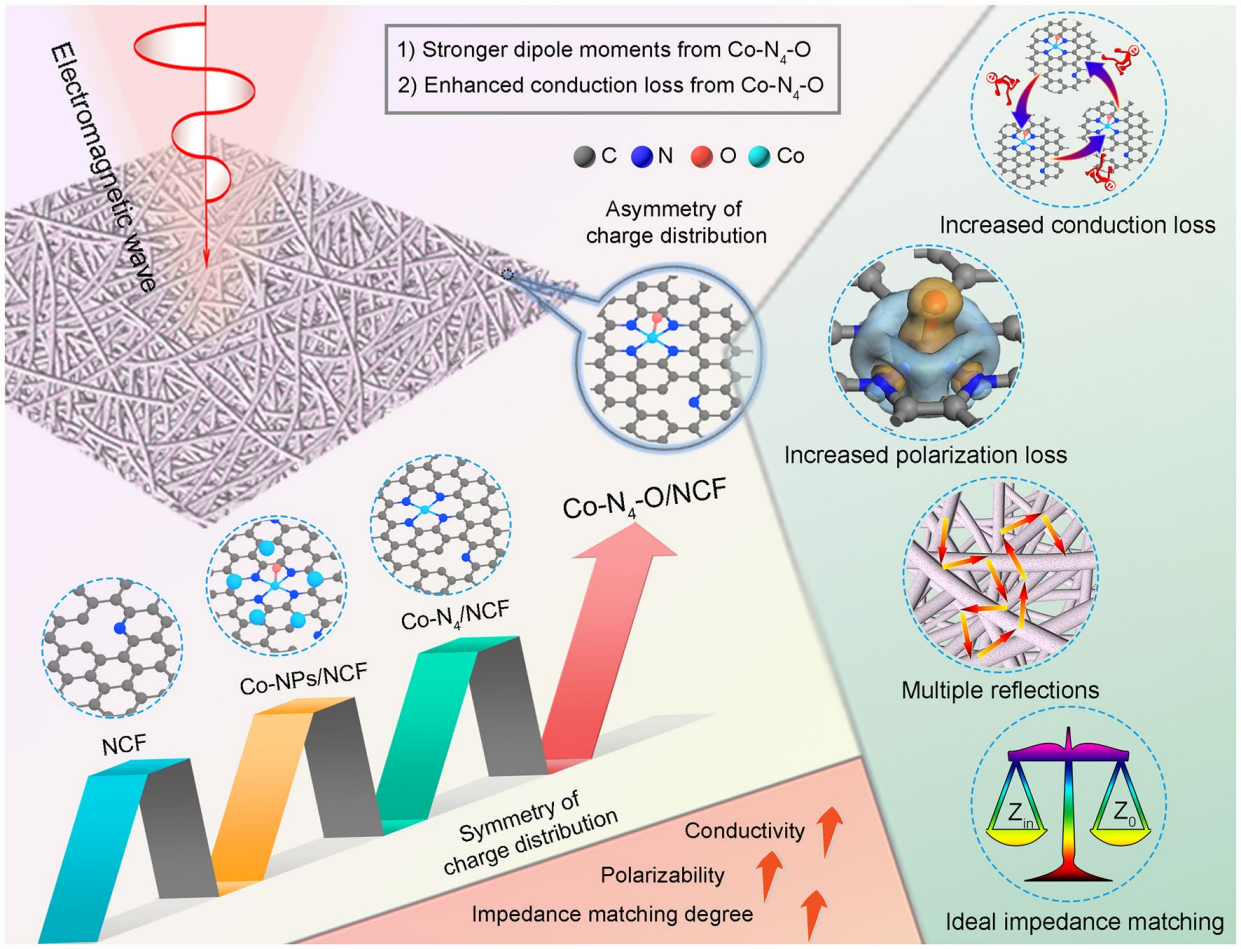
Schematic illustration of EMW absorption mechanisms for Co–N_4_–O/NCF

To further elucidate the EMW attenuation ability of the absorbers, the power loss densities of four samples were simulated using the CST Microwave Studio package. The details concerning CST microwave simulation are available in Supporting Information (Fig. S22). Figures S23 and 5g–i depict the simulated power loss densities for the NCF, Co–NPs/NCF, Co–N_4_–O/NCF, and Co–N_4_/NCF composites. The maximum power loss densities of the NCF, Co–NPs/NCF and Co–N_4_/NCF absorbers were 10.6 × 10^6^ W m^−3^ in the range of 1.5–2.4 mm, 28.6 × 10^6^ W m^−3^ in the range of 1.2–1.4 mm and 30.3 × 10^6^ W m^−3^ in the range of 2.4–2.6 mm, respectively. In contrast, the greatest power loss density of the Co–N_4_–O/NCF sample reached a value of 31.4 × 10^6^ W m^−3^ in the range of 1.2–1.4 mm. The above results indicated that Co–N_4_–O/NCF possessed the best electromagnetic energy dissipation ability, even at a comparatively small thickness.

Figure 6 summarizes the enhanced absorption mechanism of the Co–N_4_–O/NCF sample based on the findings of this study, and the main statements can be formulated as follows. (i) Compared to the planar Co–N_4_ configuration, the symmetry of the charge distribution of the asymmetric Co–N_4_–O moiety was further broken, leading to the increase in dipole moments, especially along the direction of the axial Co–O bond. Thus, the Co–N_4_–O/NCF specimen exhibited an enhancement in polarization loss compared to the Co–N_4_/NCF one, which was conducive to the improvement in the EMW absorption property of the former. (ii) Upon introduction of the axial O coordination, the DOS of the Co–N_4_–O configuration increased, thereby enhancing the conduction loss of the whole composite. (iii) The Co–N_4_–O/NCF specimen demonstrated an ideal impedance matching performance, which was due to the synergistic effect of conduction loss and polarization loss [61, 62]. Thus, most of the EMW could be absorbed by the Co–N_4_–O/NCF sample and then attenuated. (iv) The interconnected networks formed in the matrix through the wrapping among the long 1D NCF resulted in multiple reflections of the EMW, which could improve the EMW absorption property. The interconnected networks were favorable to the conduction loss of the Co–N_4_–O/NCF structure [63]. Additionally, from XPS spectra results (Figs. S6–S10), Co–N_4_/NCF and Co–N_4_–O/NCF exhibited similar species (such as C–C, C–N, C–O, C–O–O–R, graphitic–N, pyrrolic–N, Co–N_*x*_, pyridinic–N, N–O bonds, O–C–O, and C–OH), except for the Co–O species. Thus, the enhanced electromagnetic wave absorption performance was mainly attributed to the Co–N_4_–O moieties.

### Characterization and Properties of Multifunctional Film

**Fig. 7:**
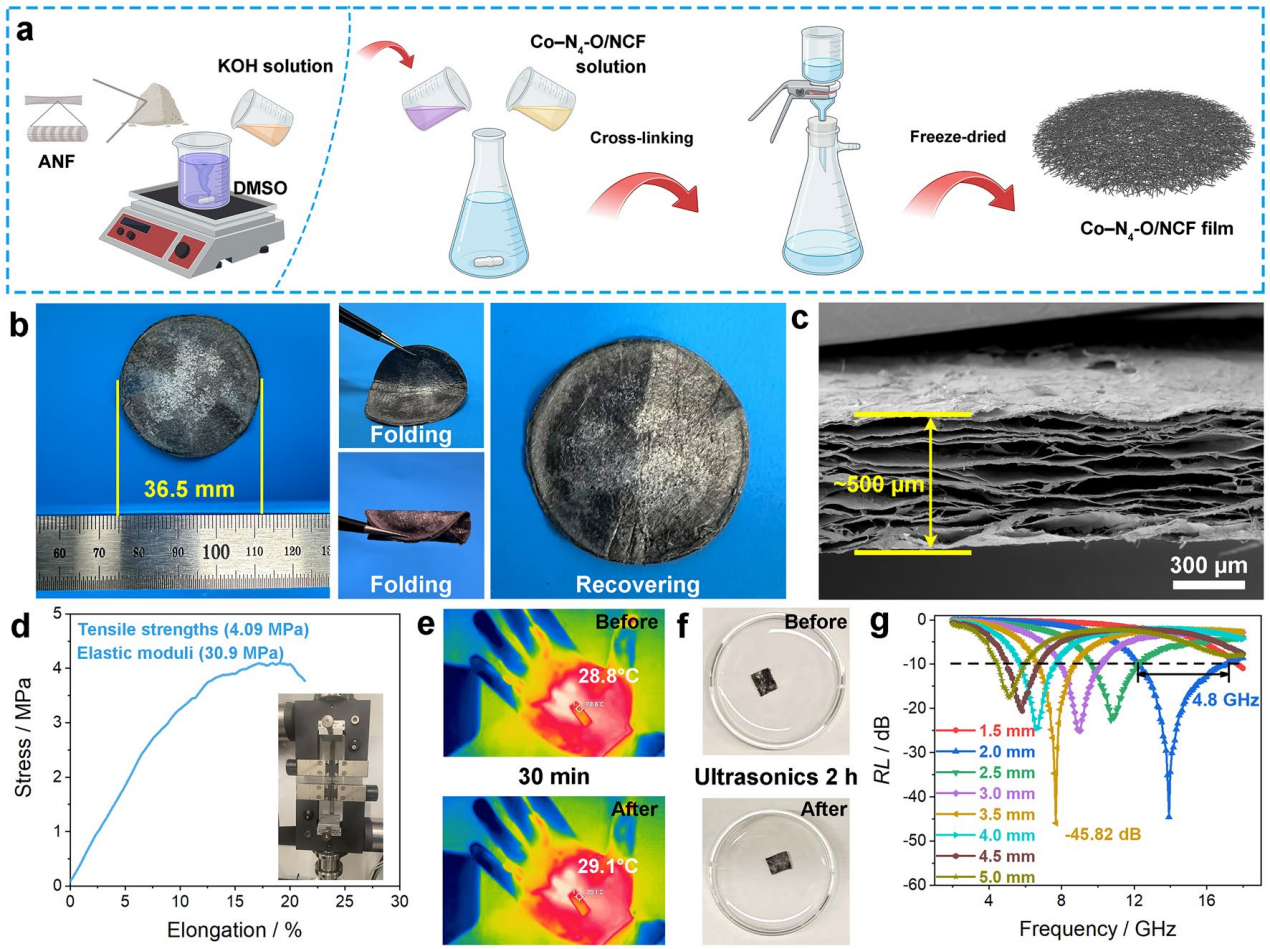
Details concerning the Co–N_4_–O/NCF film. **a** Schematic illustration of the preparation. **b** Digital photograph. **c** Structural morphology. **d** Tensile stress–strain curves. **e** Digital photos obtained with a thermal infrared imager before and after holding for 30 min. **f** Digital photos before and after ultrasonic treatment for 2 h. **g** EMW absorption performance

To benefit from the outstanding comprehensive performance of Co–N_4_–O/NCF, it is necessary to optimize the structural design of related high-performance films so as to fulfill the EMW absorption requirements in practical applications. Herein, the Co–N_4_–O/NCF film was obtained using vacuum filtration and cross-linking method, and a schematic illustration of the preparation process is depicted in Fig. 7a. It is worth mentioning that the Co–N_4_–O/NCF film demonstrated a remarkable ability to rapidly recover its original shape, i.e., good flexibility, even after undergoing multiple folding cycles (Fig. 7b). The density of the film was 0.162 g cm^−3^, implying its lightweight properties. According to the morphology image in Fig. 7c, the film thickness was approximately 500 µm, and the central layer of the cross section exhibited a fully layered structure, which promoted the enhancement of the mechanical properties. The mechanical tests of Co–N_4_–O/NCF film were conducted at a load speed of 20 mm min^−1^. According to the results (Fig. 7d), the Co–N_4_–O/NCF film possessed the outstanding tensile strength (4.09 MPa) and elastic modulus (30.9 MPa). Note that the aramid nanofiber (ANF) played a key role in enhancing mechanical, as Co–N_4_–O/NCF was uniformly dispersed in ANF. Furthermore, the defects did not significantly impact their conductivity and mechanical strength because the defect degree was same between Co–N_4_/NCF (*I*_D_/*I*_G_ value = 1.07) and Co–N_4_–O/NCF (1.07) (Fig. S11). The infrared imaging equipment was used to estimate the thermal insulation properties of the film. The Co–N_4_–O/NCF film, measuring 2.0 cm by 1.0 cm, was tested at room temperature in the hand. The digital photos of the area covered by the film displayed the yellow color (Fig. 7e). The observed temperature was steady after 30 min, suggesting that the Co–N_4_–O/NCF film possessed superior thermal insulating properties. Moreover, the structural integrity of the Co–N_4_–O/NCF film was perfectly preserved even after 2 h of ultrasonication (Fig. 7f), demonstrating its impressive stability. More importantly, the film exhibited excellent EMW absorption performance with *RL*_min_ and EAB of − 45.82 dB and 4.80 GHz at the 2.0 mm matching thickness, respectively (Figs. 7g and S24). Meanwhile, the Co–N_4_–O/NCF film exhibited huge attenuation constants and impedance matching characteristics. The radar cross-section (RCS) distribution of reflected signals at various angles is shown in Fig. S25. Specifically, the vertical reflected intensity of Co–N_4_–O/NCF film reached up to − 23.1 dB m^2^. In addition, the RCS reduction values of Co–N_4_–O/NCF film were below − 10 dB m^2^ within the range of 60° to 120°. The simulation results followed *RL* properties, demonstrating that Co–N_4_–O/NCF film held great potential as a candidate for EMW absorption. Therefore, the Co–N_4_–O/NCF film has great application potential due to its flexibility along with the outstanding mechanical properties, thermal insulating feature, stability, and high-performance EMW absorption properties.

## Conclusions

In summary, the atomically dispersed Co–N_4_ moiety in coordination with an axial direction oxygen atom supported on the nitrogen-doped carbon nanofiber (Co–N_4_–O/NCF) was successfully prepared via a facile water-assisted carbonization strategy. According to theoretical calculations, introducing the axial Co–O coordination made the charge distribution symmetry break near Co atoms, which significantly strengthened the polarization loss, especially along the coordination itself. Meanwhile, the axial O atoms also increased the conduction loss of the Co–N_4_–O/NCF system. As a result, excellent electromagnetic wave absorption performance of the Co–N_4_–O/NCF film with a high reflection loss of − 45.82 dB and the effective absorption bandwidth of 4.8 GHz at the matching thickness of 2.0 mm was achieved, exceeding those of the planar symmetrical Co–N_4_ counterpart. Simultaneously, the film displayed light weight, flexibility, excellent mechanical properties, great thermal insulating feature, and superior stability. This work highlights the importance of coordination environments of single atoms to the dielectric and electromagnetic wave absorption properties, opening up new prospects for designing multifunctional high-performance metal single-atom-based absorbers.

## Supplementary Information

Below is the link to the electronic supplementary material.

Supplementary file1 (PDF 3858 KB)
