# Supplementary material for: Multifunctional Film Assembled from N-Doped Carbon Nanofiber with Co–N4–O Single Atoms for Highly Efficient Electromagnetic Energy Attenuation
Source: Nanomicro Lett. 2024 Jul 9;16:240. doi: 10.1007/s40820-024-01440-2 (PMC11233488; doi:10.1007/s40820-024-01440-2)
Supplement: Supplementary file 1 — Supplementary file1 (PDF 3858 KB) [file 40820_2024_1440_MOESM1_ESM.pdf]

Supporting Information for

Multifunctional Film Assembled from N-Doped Carbon Nanofiber with Co-N_4-O Single Atoms for Highly Efficient Electromagnetic Energy Attenuation

Jia Xu[1, 2, #], Bei Li[2, #], Zheng Ma[1], Xiao Zhang[2], Chunling Zhu[1, *], Feng Yan[2, *], Piaoping Yang[1] and Yujin Chen[1, 2, *]

[1] College of Materials Science and Chemical Engineering, Harbin Engineering University, Harbin 150001, P. R. China

[2] Key Laboratory of In-Fiber Integrated Optics, College of Physics and Optoelectronic Engineering, Harbin Engineering University, Harbin 150001, P. R. China

# Jia Xu and Bei Li contributed equally to this work.

*Corresponding authors. E-mail: chenyujin@hrbeu.edu.cn (Yujin Chen); yanfeng@hrbeu.edu.cn (Feng Yan); zhuchunling@hrbeu.edu.cn (Chunling Zhu)

S1 Calculation Formulas of Electromagnetic Properties

S1.1 The conduction loss (ε_c'') and polarization loss (ε_p''). Based on Debye theory, the relatively complex permittivity imaginary part (ε'') is deduced from:

$$\varepsilon'' = \varepsilon_p'' + \varepsilon_c'' = \frac{\varepsilon_s - \varepsilon_\infty}{1+\omega^2\tau^2}\omega\tau + \frac{\sigma}{\omega\varepsilon_0} \quad \text{(S1)}$$

$$\varepsilon_p'' = \frac{\varepsilon_s - \varepsilon_\infty}{1+\omega^2\tau^2}\omega\tau \quad \text{(S2)}$$

$$\varepsilon_c'' = \frac{\sigma}{\omega\varepsilon_0} \quad \text{(S3)}$$

where, ε'' is the imaginary part of the complex permittivity, ε_∞ is the optical permittivity, ε_s is the static permittivity, ω is the angular frequency, τ is the relaxation time for polarization, σ is the electrical conductivity, ε_0 is the vacuum permittivity (8.85×10^{-12} F/m).

S1.2 The reflection loss (*RL*). The *RL* of absorbers can be calculated by utilizing the relative complex permittivity (ε_r) and permeability (μ_r),

$$RL(dB) = 20\lg\left|\frac{Z_{in}-Z_0}{Z_{in}+Z_0}\right| \quad \text{(S4)}$$

$$Z_{in} = Z_0\sqrt{\frac{\mu_r}{\varepsilon_r}}\tanh\left[\frac{2j\pi fd}{c}\sqrt{\mu_r\varepsilon_r}\right] \quad \text{(S5)}$$

where ε_r is the relative complex permittivity, μ_r is the relative complex permeability, Z_0 is the impedance of free space, Z_{in} is the input characteristic impedance, f is the frequency of electromagnetic wave, d is the absorber thickness, and c is the velocity of light (3.0×10^8 m s^{-1}), respectively.

S1.3 The attenuation constant (α). The α can be obtained by:

$$\alpha = \frac{\omega}{\sqrt{2}c}\sqrt{\varepsilon'\mu'\left[\frac{\varepsilon''}{\varepsilon'}\frac{\mu''}{\mu'} - 1 + \sqrt{\left(1+\left(\frac{\varepsilon''}{\varepsilon'}\right)^2\right)\left(1+\left(\frac{\mu''}{\mu'}\right)^2\right)}\right]} \quad \text{(S6)}$$

S1.4 The impedance matching degree (M_z) can be given as:

$$M_z = \frac{2M_{in}'}{|Z_{in}|^2+1} \quad \text{(S7)}$$

where Z_{in}' refers to the real part of Z_{in}. The optimal impedance matching of an absorber is achieved at $M_Z \rightarrow 1$.

S2 Supplementary Figures and Tables

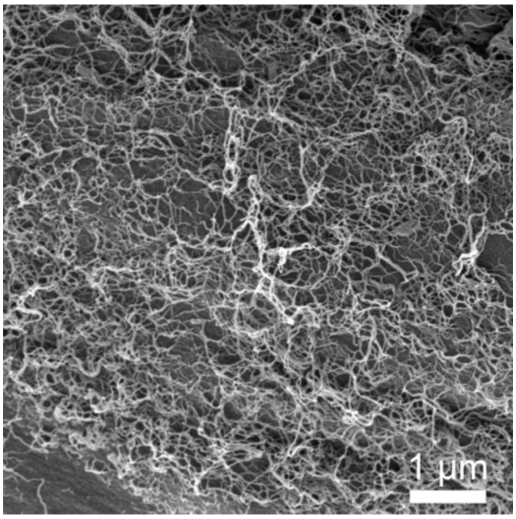

Fig. S1 SEM image of NCF

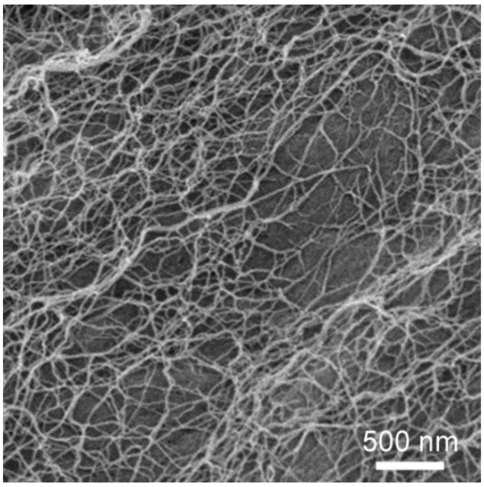

Fig. S2 SEM image of Co–NPs/NCF

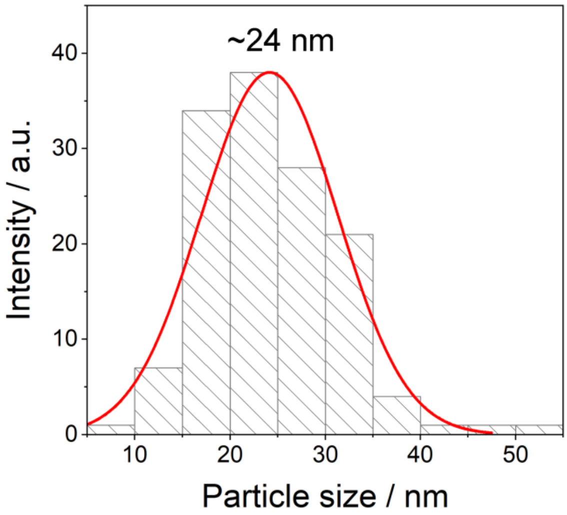

Fig. S3 The distribution diagram of Co NPs diameter of Co–NPs/NCF

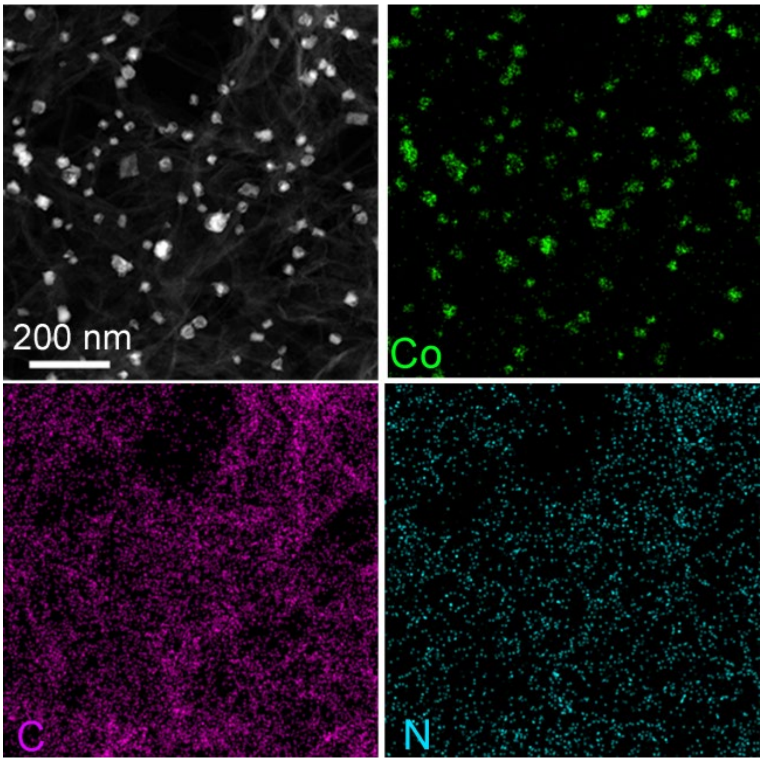

Fig. S4 HAADF–STEM image of Co–NPs/NCF and corresponding elemental maps

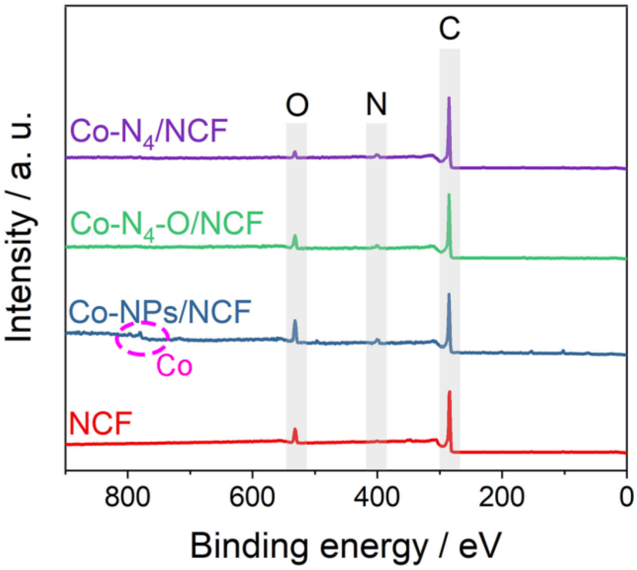

Fig. S5 XPS survey spectra of NCF, Co–NPs/NCF, Co–N_4–O/NCF and Co–N_4/NCF

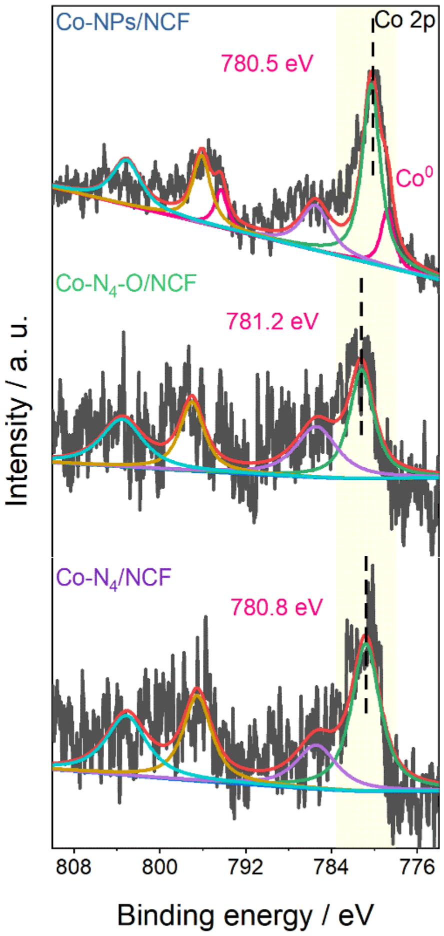

Fig. S6 The high–resolution Co 2*p* XPS spectra of Co–NPs/NCF, Co–N_4–O/NCF and Co–N_4/NCF

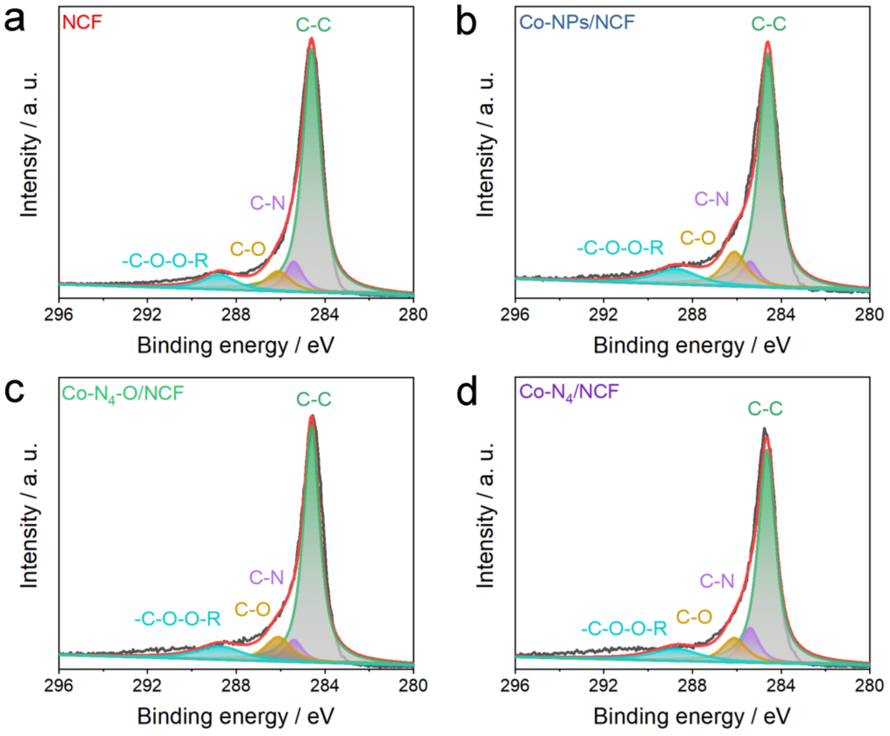

Fig. S7 The high–resolution C 1*s* XPS spectra of (**a**) NCF, (**b**) Co–NPs/NCF, (**c**) Co–N_4–O/NCF and (**d**) Co–N_4/NCF

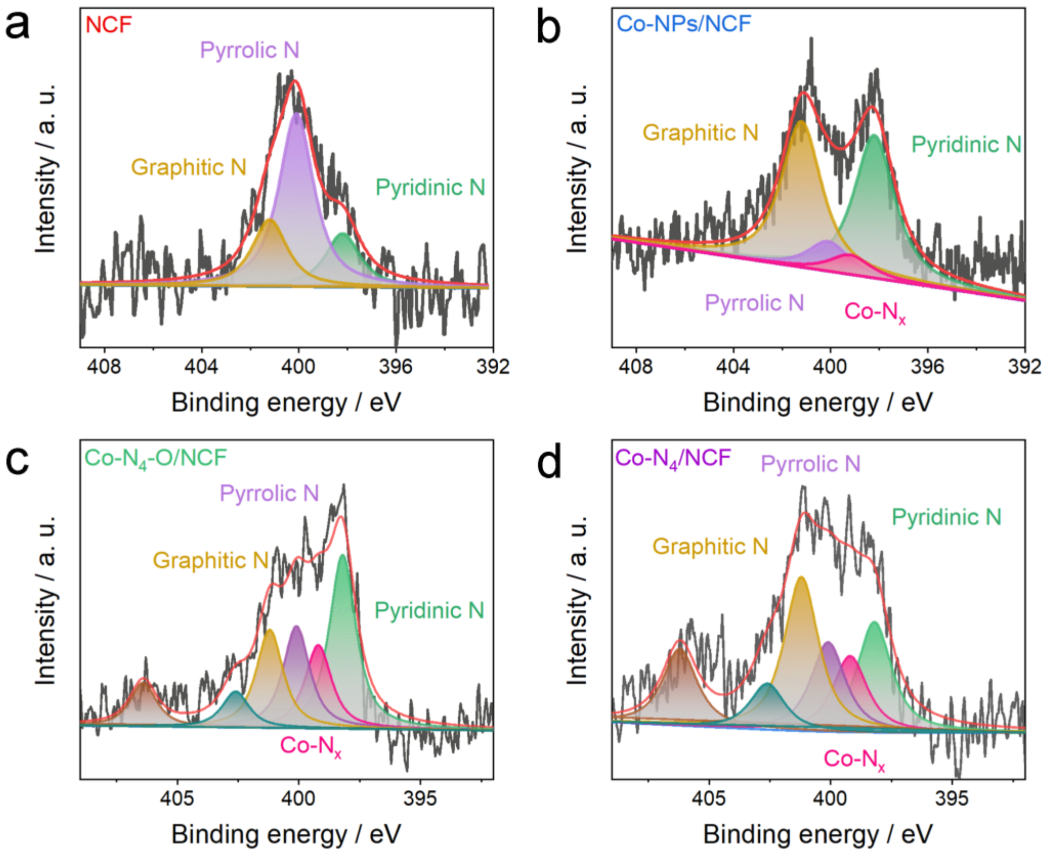

Fig. S8 The high–resolution N 1*s* XPS spectra of (**a**) NCF, (**b**) Co–NPs/NCF, (**c**) Co–N_4–O/NCF and (**d**) Co–N_4/NCF

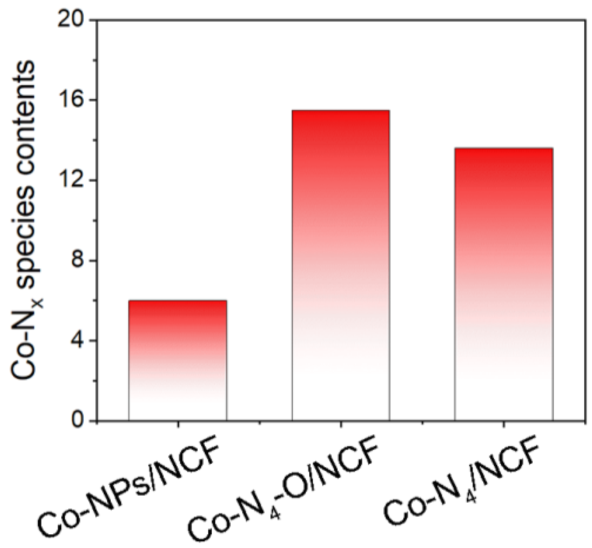

Fig. S9 Co-N_x contents of Co–NPs/NCF, Co–N_4–O/NCF and Co–N_4/NCF

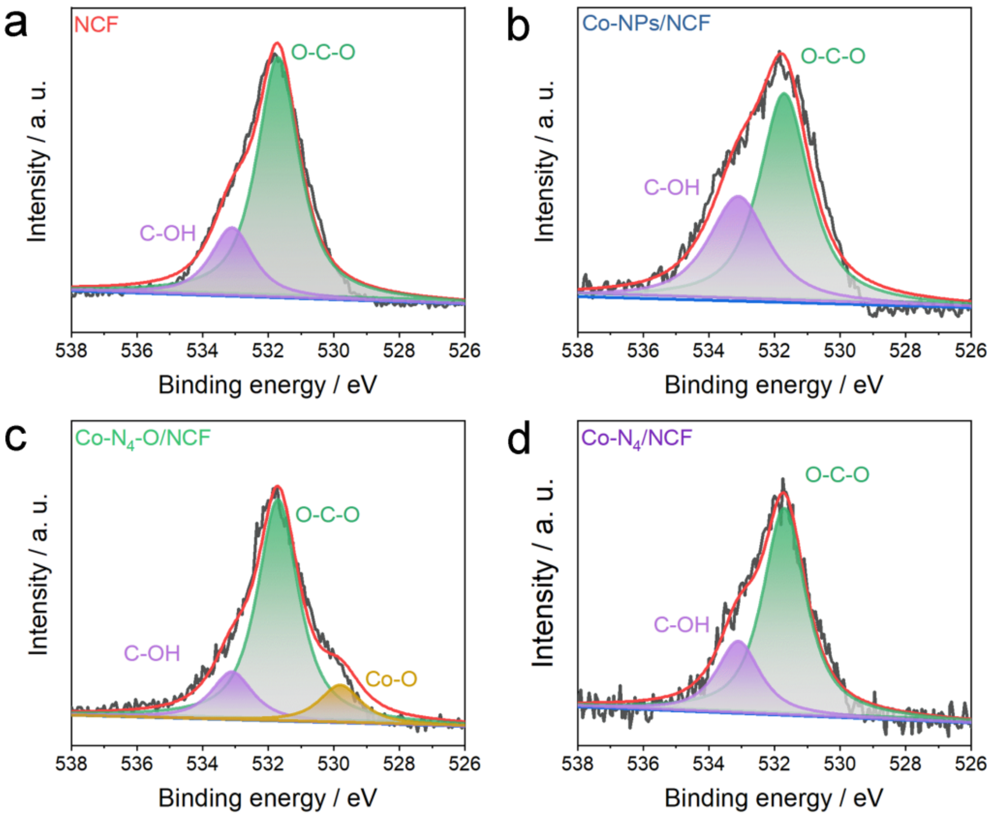

Fig. S10 The high–resolution O 1*s* XPS spectra of (**a**) NCF, (**b**) Co–NPs/NCF, (**c**) Co–N_4–O/NCF and (**d**) Co–N_4/NCF

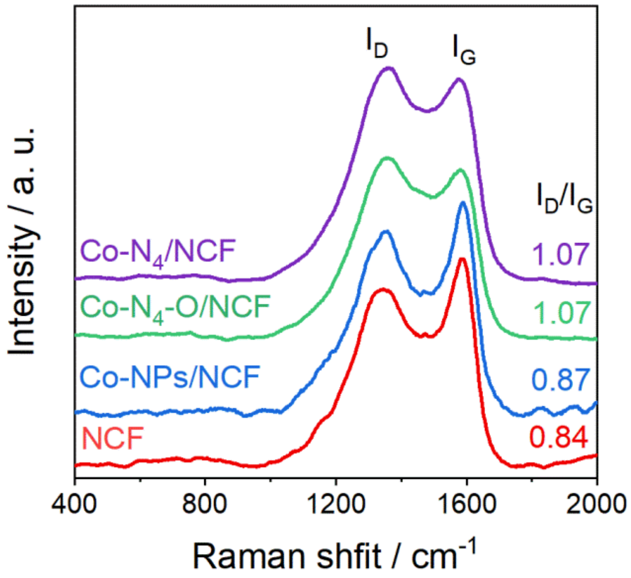

Fig. S11 Raman spectra of NCF, Co–NPs/NCF, Co–N_4–O/NCF and Co–N_4/NCF

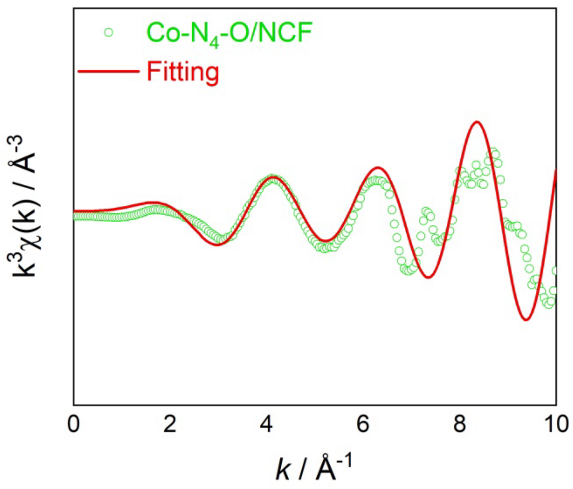

Fig. S12 The EXAFS fitting curves of Co–N_4–O/NCF at *r* space

Table S1 EXAFS fitting parameters of sample and at the Co K-edge (S_0^2=0.77)

Sample	Path	C.N.	R (Å)	$\sigma^2 \times 10^3$ (Å^2)	ΔE (eV)	R factor
**Co foil**	Co–Co	12	2.49±0.01	6.2±0.1	7.6±0.2	0.001
**Co_3O_4**	Co–O	4.2±0.4	1.92±0.01	1.9±0.8	2.3±1.3	0.006
	Co–Co	4.4±1.8	2.88±0.02	4.1±2.6	2.5±3.2	
	Co–O	5.0±2.6	3.34±0.02	3.2±3.0	-2.8±3.4	
**CoPc**	Co–N	4.1±0.6	1.91±0.01	2.5±0.8	-5.2±3.1	0.017
	Co–C	6.9±2.1	3.00±0.02	2.6±1.7	6.9±3.3	
**Co–N_4–O/NCF**	Co–N	4.0±0.2	1.96±0.03	2.2±2.2	-4.1±2.6	0.017
	Co–O	1.0±0.1	1.71±0.05			
**Co–N_4/NCF**	Co–N	3.9±0.2	1.92±0.02	19.0±4.2	-8.8±3.0	0.009

C.N.: coordination numbers; *R*: bond distance; σ^2: Debye-Waller factors; Δ*E*: the inner potential correction.

R factor: goodness of fit.

* fitting with fixed parameter.

S_0^2 was set to 0.77 according to the experimental EXAFS fit.

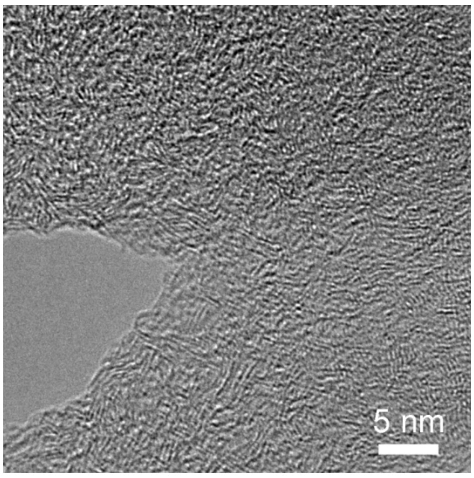

Fig. S13 HRTEM image of Co–N_4/NCF

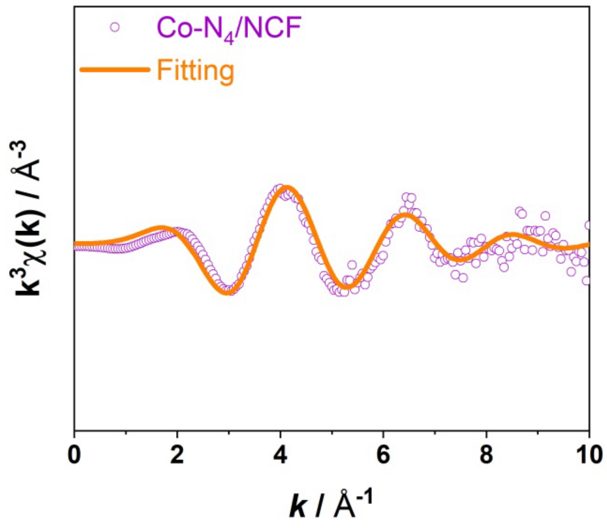

Fig. S14 EXAFS fitting curves of Co–N$_4$/NCF at *r* space

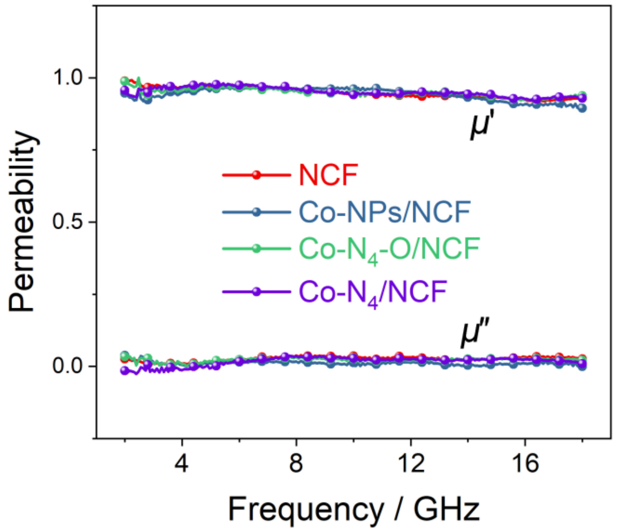

Fig. S15 μ'–f and μ''–f curves of NCF, Co–NPs/NCF, Co–N$_4$–O/NCF and Co–N$_4$/NCF

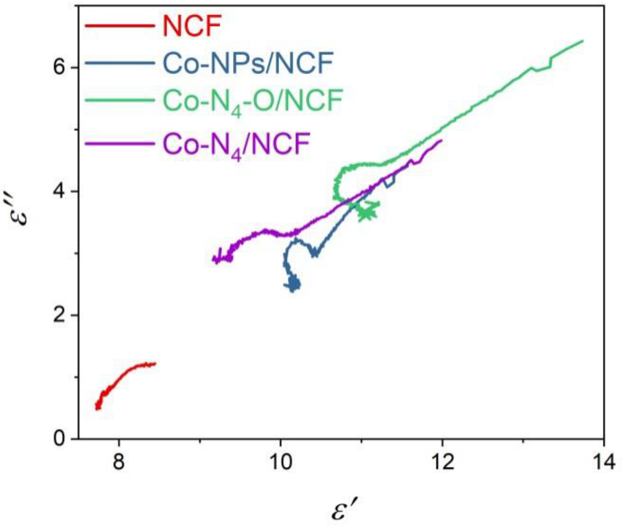

Fig. S16 Cole–Cole plots of NCF, Co–NPs/NCF, Co–N$_4$–O/NCF and Co–N$_4$/NCF

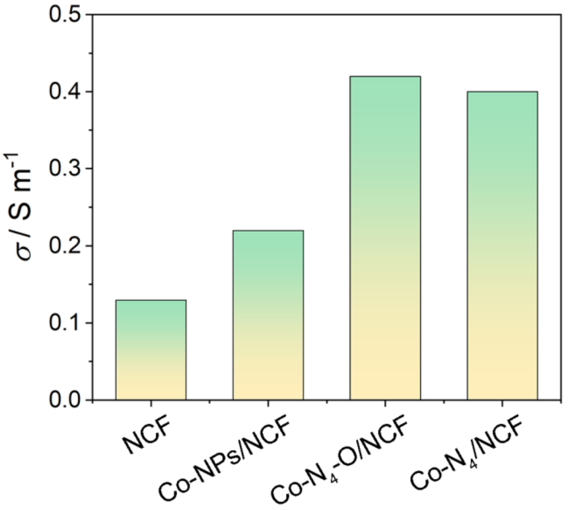

Fig. S17 *σ* of NCF, Co–NPs/NCF, Co–N_4–O/NCF and Co–N_4/NCF

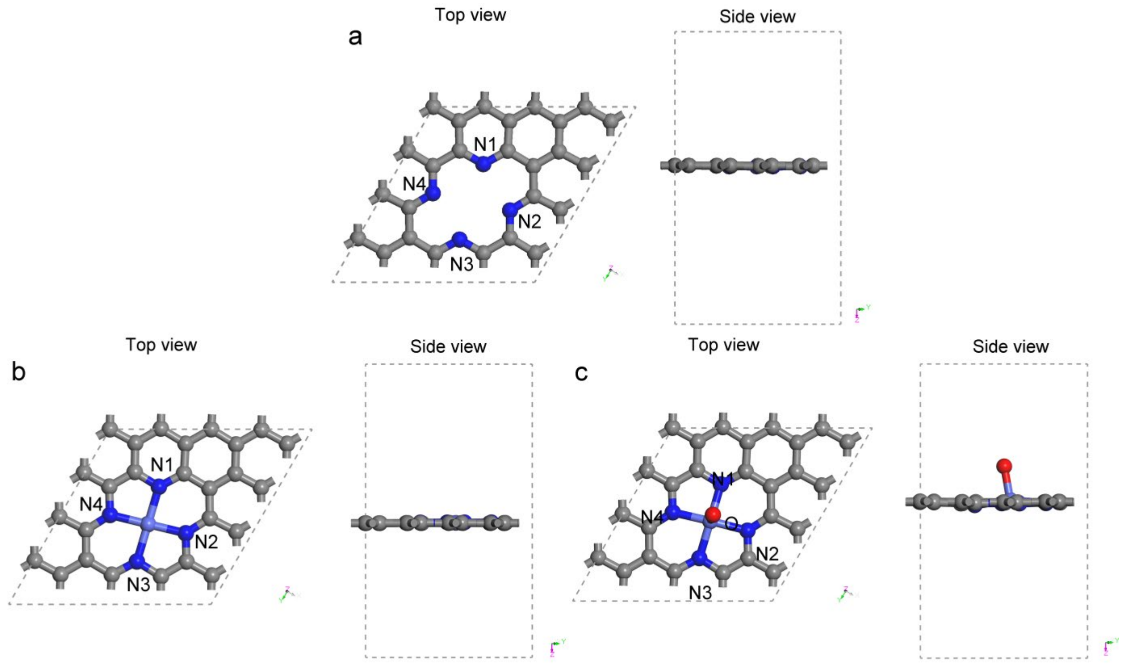

Fig. S18 Top and side views of (**a**) C–N, (**b**) Co–N_4 and (**c**) Co–N_4–O configurations

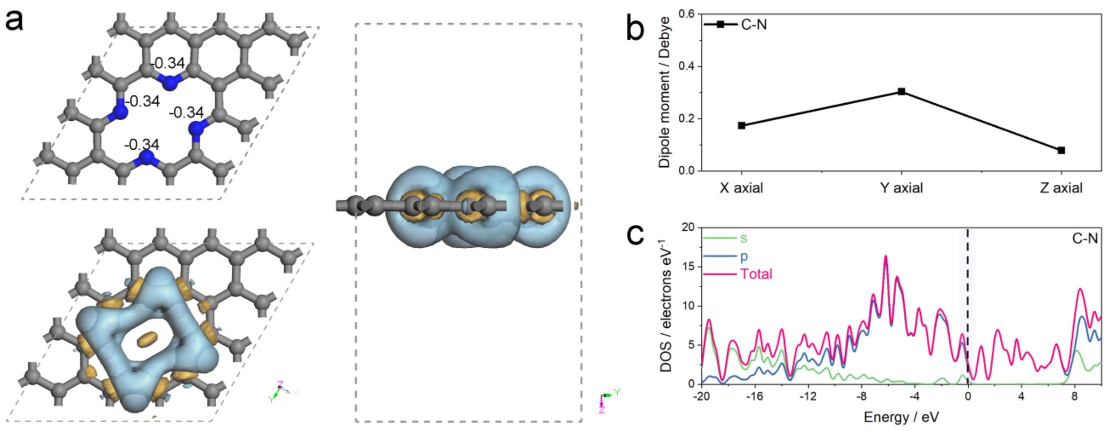

Fig. S19 (**a**) Mulliken population distributions and the charge density difference of C-N configurations. (**b**) Dipole moment values of C–N configurations. (**c**) The calculated projected density of states (DOSs) of C–N configurations

Table S2 Mulliken charges of Co, O, and N atoms in the C-N, Co–N_4 and Co–N_4–O configurations

Configuration	Elements	Mulliken charge (e)
**Co–N_4**	Co	+1.13
	N1, N2, N3, N4	-0.45, -0.45, -0.45, -0.45
**Co–N_4–O**	Co	+1.49
	N1, N2, N3, N4, O	-0.43, -0.44, -0.46, -0.44, -0.55
**C-N**	N1, N2, N3, N4	-0.34

Table S3 Dipole moment values of C-N, Co–N_4 and Co–N_4–O configurations

Configuration	Co–N_4	Co–N_4–O	C-N
**X-axial**	0.17569	0.21091	0.17345
**Y-axial**	0.30327	0.33904	0.30306
**Z-axial**	0.07833	0.37087	0.07833

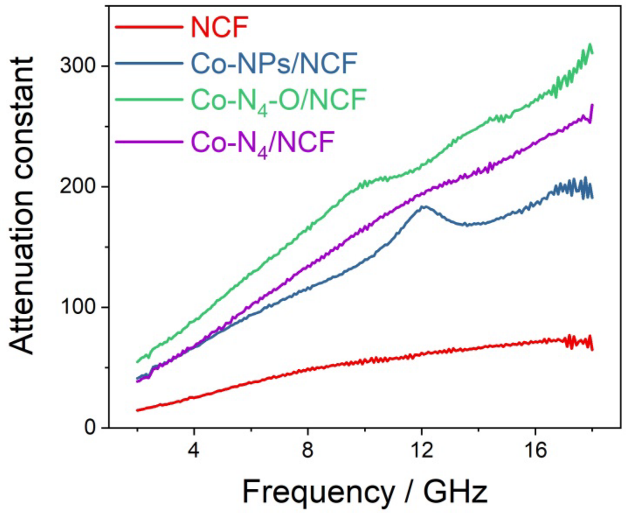

Fig. S20 The attenuation constants of NCF, Co–NPs/NCF, Co–N_4–O/NCF and Co–N_4/NCF

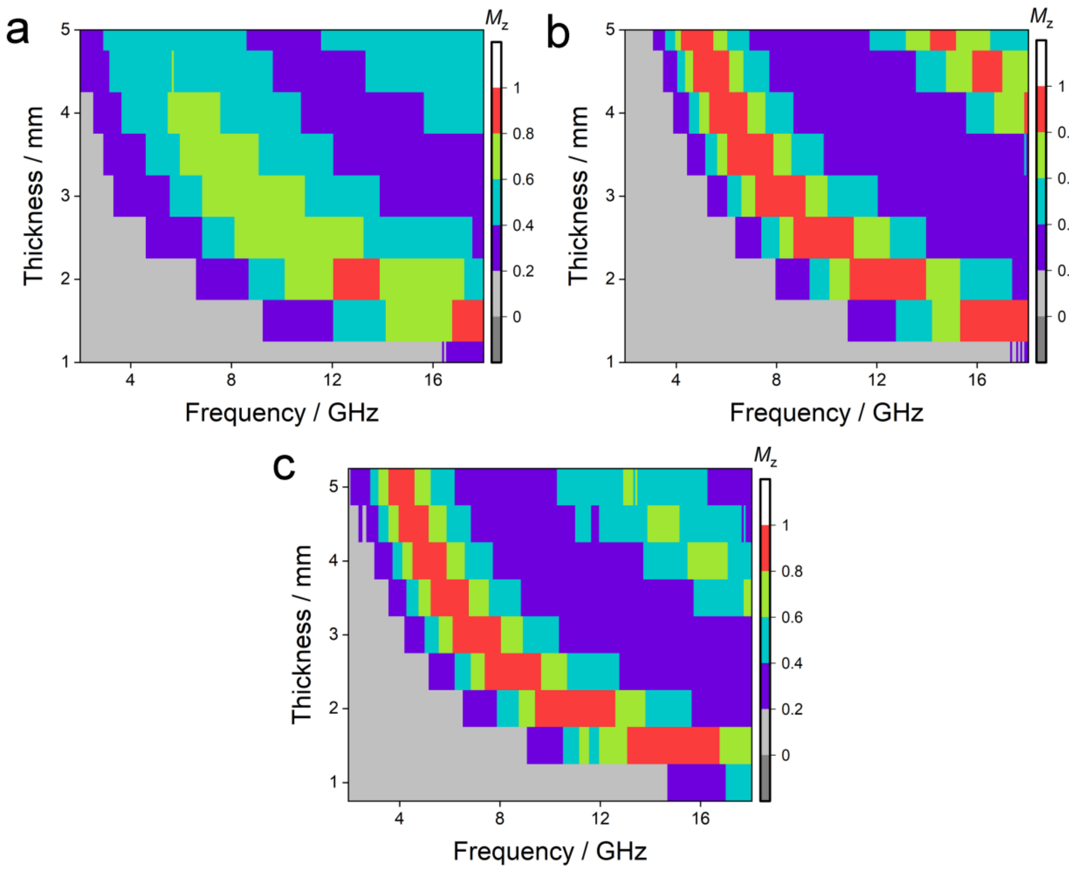

Fig. S21 The impedance matching characteristics of (**a**) NCF, (**b**) Co–NPs/NCF and (**c**) Co–N_4/NCF

Table S4 Comparison of EMW absorption performance

Absorbers	*RL* / dB	d / mm	EAB_{10} / GHz	Filler / %	Refs
**Co-N_4-O/NCF**	**–41.32**	**1.5**	**4.02**	**10**	**This work**
**Co-N_4-O/NCF film**	**–45.82**	**2.0**	**4.80**	**10**	**This work**
**CMT@CNT/Co**	−52.3	2.0	5.1	15	Adv. Funct. Mater. **2019,** 29, 1901448.
**Mo_2N@CoFe@C/CNT**	−53.5	2.0	5.0	20	Nano-Micro Lett. **2021,** 13(1), 1-15.
**Zn/Co hybrid materials**	-45.85	1.6	4.8	33.3	Adv. Funct. Mater. **2021,** 2106677.
**1T phase MoS_2**	−45.9	3.5	3.89	50	Adv. Funct. Mater. 2021, 31, 2011229.
**NbS_2**	−43.85	2.0	6.48	40	Adv. Funct. Mater. **2021,** 2108194.
**TiO_2/$ZrTiO_4$**	−67.8	2.7	5.9	35	Nano-Micro Lett. **2021,** 13(1):1-16.
**CoS_2@MoS_2**	−58	3.0	6.24	20	Chem. Eng. J. **2019,** 378, 122159.
**ZnO/OMCS**	−39.3	2.0	9.1	30	Nano-Micro Letters **2021,** 13(1).
**Co@NPC**	−51.2	1.62	4.4	25	Chem. Eng. J. **2018,** 339, 432-441.

**NiCo@C/ZnO**	−60.9	2.3	6.8	33.3	Nano-Micro Lett. **2021**, 13, 175.
**Co@NC-ZnO**	−69.6	2.4	6.8	0.25	Small **2021**, 17, 2100970.
**Sn@Mo_2C/C**	−52.1	3.5	6.76	30	Small **2021**, 17, 2100283.
**C/MoS_2**	−50.1	2.4	6	40	Nano-Micro Lett. **2021**, 13, 43.
**FeNC@rGO**	−40.2	2.5	3.9	20	Appl. Phys. Lett. **2020,** 116, 153101.
**Co_xNi_y@C**	−43.7	1.7	5.7	20	Nano-Micro Lett. **2020**, 12, 102.
**Ni-SAs3/NC**	−57.8	2.5	7.08	45	Adv. Funct. Mater.**2023**, 33, 2212604.
**Co_{1+Cs}/NGC**	−54.3	2.0	7.0	6.0	Adv. Funct. Mater. **2023**, 33, 2304442.
**Ni-SA/HPCF**	−53.0	3.5	5.0	10	Adv. Funct. Mater. **2023**, 33, 2210456

S3 Electromagnetic Simulation

The power loss density simulation in the range of 2-18 GHz was implemented by Computer Simulation Technology (CST) Microwave Studio. The boundary conditions were applied with the electric field along the *y* direction and the magnetic field along the *z* direction. Open (add space) boundary conditions were used in all directions. The model's width was 200 × 200 mm^2, the absorber's thickness was 1.5 mm, and the PEC's thickness was 1.0 mm.

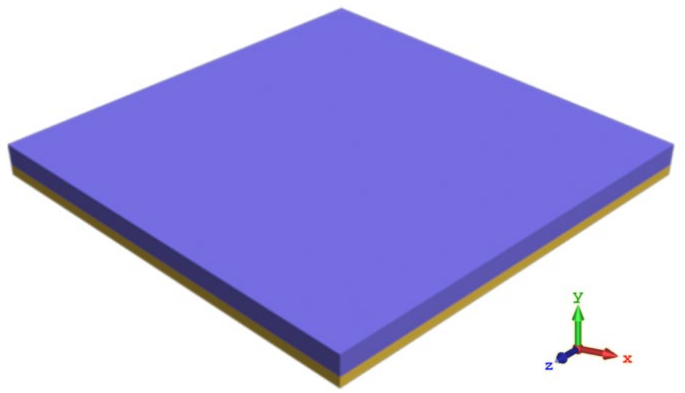

Fig. S22 The model of CST simulation

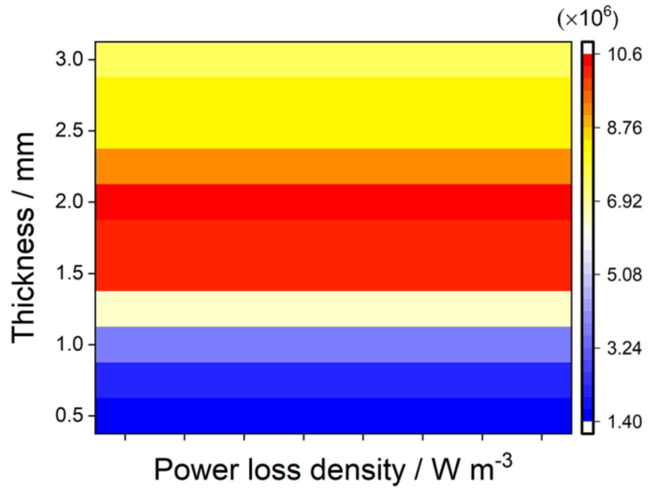

Fig. S23 The power loss densities of NCF

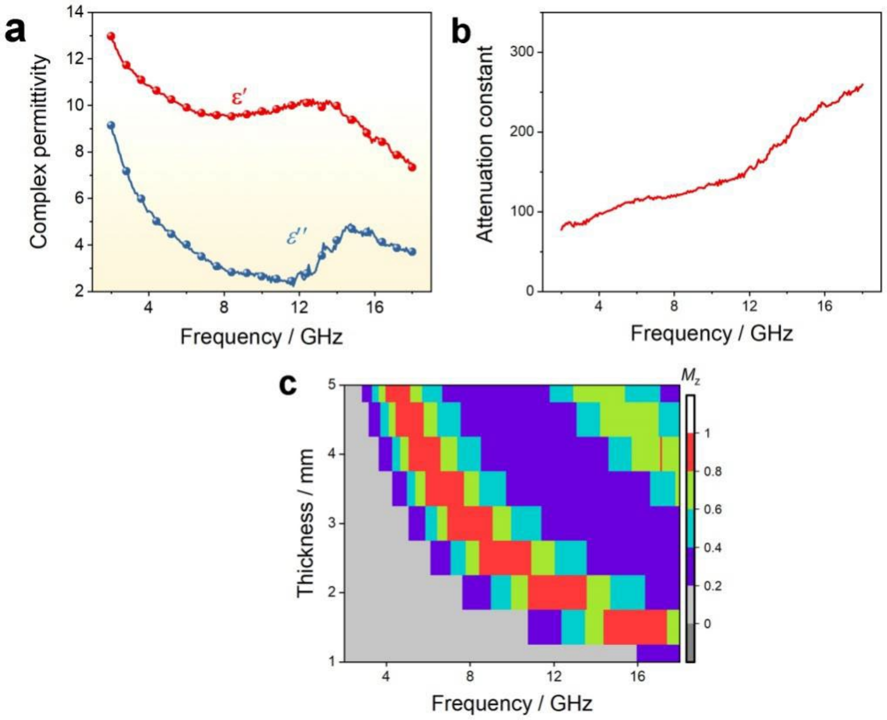

Fig. S24 (**a**) The complex permittivity, (**b**) the attenuation constants and (**c**) the impedance matching characteristics of Co–N_4–O/NCF film

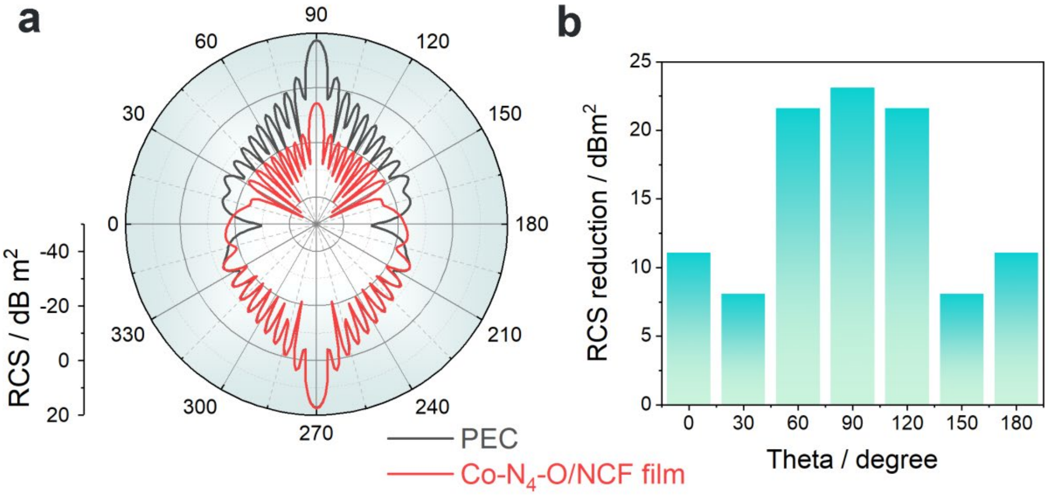

Fig. S25 (**a**) The polar coordinate diagram and (**b**) RCS reduction of Co-N_4-O/NCF film
